# Pure-Blue Emission at the Edge of the CIE Diagram: Carbazolyl NHC Platinum Butterfly Complexes with Near-Unity Quantum Yield and Their Application in Organic Light-Emitting Diodes

**DOI:** 10.1021/acs.inorgchem.6c00582

**Published:** 2026-05-01

**Authors:** Jorge Roy, Sergio Martínez-Saiz, Michele Forzatti, Antonio Martín, Daniel Escudero, Daniel Tordera, Violeta Sicilia

**Affiliations:** † Departamento de Química Inorgánica, Facultad de Ciencias, Instituto de Síntesis Química y Catálisis Homogénea (ISQCH), CSIC—Universidad de Zaragoza, Pedro Cerbuna 12, 50009 Zaragoza, Spain; ‡ Instituto de Ciencia Molecular, Universidad de Valencia, C/Catedrático J.Beltran, 2, 46980 Paterna, Spain; § Department of Chemistry, KU Leuven, Celestijnenlaan 200f—Box 2404, 3001 Leuven, Belgium; ∥ Departamento de Química Inorgánica, Escuela de Ingeniería y Arquitectura de Zaragoza, Instituto de Síntesis Química y Catálisis Homogénea (ISQCH), CSIC—Universidad de Zaragoza, Campus Río Ebro, Edificio Torres Quevedo, 50018 Zaragoza, Spain

## Abstract

The Pt butterfly complexes, [{Pt­(Cz-C^∧^C*_im_)­(μ-Rpz)}_2_] (HC^∧^C* = 1-(4-(carbazolyl) phenyl)-3-methyl-1*H*-imidazol-2-ylidene; Rpz = pyrazolate (pz) **1**; 4-fluoropyrazolate (4-Fpz) **2**; 4-trifluoromethylpyrazolate (4-CF_3_pz) **3**), with a carbazole-appended cyclometalated N-heterocyclic carbene (Cz-C^∧^C*_im_) in the wings, were prepared and characterized. DFT and TD-DFT calculations show the prevalent ^1/3^ILCT character of the lowest-energy absorptions and emissions of these phosphorescent complexes. In 5 wt % PMMA films, complexes **1**–**3** exhibit blue phosphorescence with photoluminescence quantum yield up to 0.99, even in air. The Commission Internationale de L’Eclairage (CIE) coordinates of their emission are in the blue corner, being “pure-blue” (CIE_*x*+*y*_ < 0.3) in the case of **1**. In view of these properties, these complexes were used to prepare OLEDs, with the best-performing devices achieving a maximum external quantum efficiency of 4.26% and maximum luminance of 2357.7 cd m^–2^ with cyan electroluminescence (CIE_*x*,*y*_ = 0.14, 0.46).

## Graphical abstract

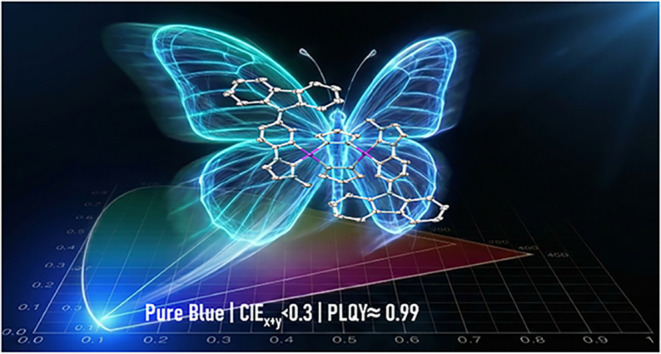

## Introduction

Phosphorescent transition-metal complexes have emerged as a pivotal class of functional materials, owing to their outstanding photophysical properties and versatile applications.^1^ Among them, phosphorescent and phosphorescent sensitized thermally assisted delayed fluorescent organic light-emitting diodes (PhOLEDs and PS-TADF OLEDs),^2^ which incorporate a third-row transition-metal phosphorescent complex, predominantly those of Ir­(III) or Pt­(II), in the light-emitting layer (EML) as an active emitter or sensitizer, respectively.

The high spin–orbit coupling (SOC) constant of Ir­(III) and Pt­(II) promotes efficient intersystem crossing (ISC) from the lowest singlet (S_1_) to the lowest triplet (T_1_) excited states of the complex and fast decay from T_1_ to the ground state (GS). This feature allows harvesting triplet excitons, in addition to singlet ones, when they are incorporated into the emitting layer of the OLEDs, raising the internal quantum efficiency (IQE) from 25% to 100% ^3–6^ and the luminance level compared to that of their “purely organic” counterparts.

Therefore, PhOLEDs and PS-TADF OLEDs^2^ are at the forefront of light-emitting technology, such as virtual reality displays and automotive panels, which demand higher luminance levels, efficiency, and stability than those provided by fluorescent OLEDs. Among the primary colors, the development level of pure-blue PhOLEDs in terms of efficiency and stability does not match that of red and green devices and remains a challenge. To this end, it is necessary to search for efficient and stable phosphors with pure-blue emission and high color purity.^6^

In the chemistry of Pt­(II), many mononuclear complexes have been probed in these kinds of devices.^7–13^ To a lesser extent, dinuclear complexes with different architectures have also been tested.^14,15^ Among them are those known as *platinum butterfly* complexes: dinuclear cyclometalated complexes with bridging pyrazolate groups, formula [{Pt­(C^∧^E)­(μ-Rpz)}_2_] (E = N, C_NHC_) (see Scheme 1, parts a,^16^ b,^17^ c,^18^ d,^14^ e^2^).

**Scheme 1:**
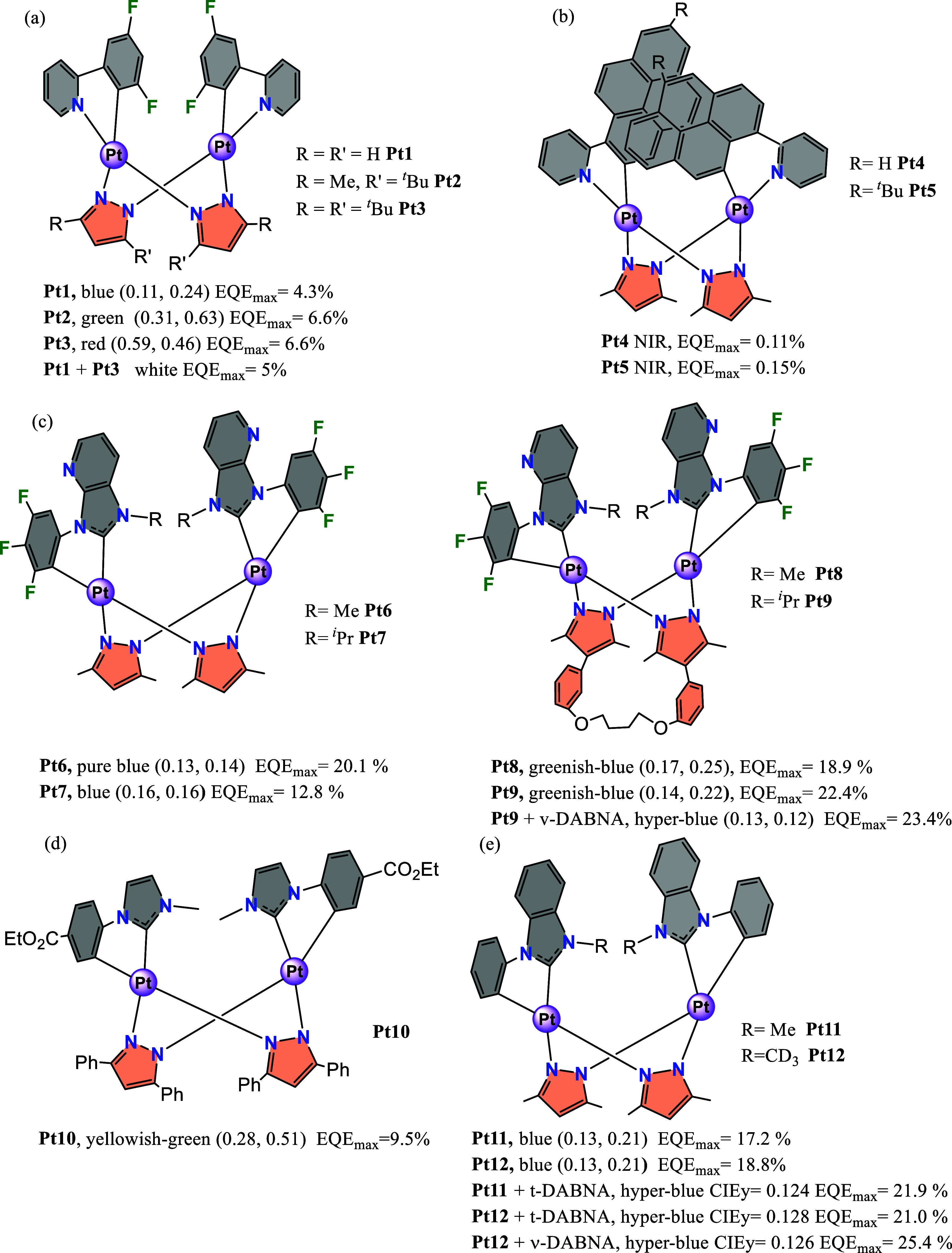
Pt-Butterfly Complexes Used in OLEDs Represented in Parts (a)–(e)

*Platinum butterfly* complexes with C^∧^N ligands in the wings were first studied by Castellano,^19,20^ Thompson,^16,21^ Ma^22^ et al. They found that the intramolecular Pt···Pt distance shortens and the metallophilic interaction strengthens as the bulkiness of 3,5-disubstituted pyrazolate groups (butterfly body) increases, changing the nature of the emissive state from ^3^IL/^3^MLCT to ^3^MMLCT. Therefore, the emission color can be smartly tuned from blue to red by both selecting the C^∧^N group and controlling the intermetallic distance *via* the bulkiness of the bridging pyrazolate. *Platinum butterfly* complexes with C^∧^C_NHC_ (C^∧^C*) ligands in the wings were studied by the groups of Strassner,^23,24^ Che,^18^ Sicilia,^14,25^ and Kim.^2^ Cyclometalated C^∧^C* groups induce greater field splitting than C^∧^N ones, raising the d_x2–y2_ antibonding orbital and preventing the population of ^3^MC states. Since the population of this orbital causes severe structural distortion and provides an easy nonradiative pathway,^1^ complexes with a “Pt^II^(C^∧^C*)” chromophore unit show very high photoluminescence quantum yields (PLQYs).^26,27^

In the course of our research, we prepared complexes [{Pt­(EtO_2_C-C^∧^C*)­(μ-Rpz)}_2_] (HC^∧^C* = 1-(4-(ethoxy-carbonyl)­phenyl)-3-methyl-1*H*-imidazol-2-ylidene; Rpz = pyrazolate (pz); 4-methylpyrazolate (4-Mepz); 3,5-dimethylpyrazolate (3,5-Me_2_pz); and 3,5-diphenylpyrazolate (3,5-Ph_2_pz)).^25^ They exhibit two conformers in the ground state (GS), the *butterfly spread* and the *butterfly folded* ones, characterized by long and short Pt–Pt distances, respectively. These conformers interconvert via an intramolecular flappinglike motion, which is barrierless in compounds with bulkier 3,5-disubstituted pyrazolates. Nondoped OLEDs with [{Pt­(EtO_2_C-C^∧^C*)­(μ-3,5-Ph_2_pz)}_2_] (**Pt10**, in Scheme 1d) were fabricated.^14^ The EL of devices with a thick layer (10 nm) matches the emission of **Pt10** due to a ^3^MMLCT state of butterfly folded molecules with short Pt–Pt distances, while that of devices with an ultrathin layer (1 nm) shows a higher contribution of emission from the ^3^IL/MLCT state of butterfly spread molecules with long Pt–Pt distances. These changes in the structural conformation, induced by the thickness of the layer, are reflected in the CIE (International Commission on Illumination) color coordinates (0.38, 0.57) and (0.28, 0.51) for the thick and ultrathin devices, respectively. The ultrathin OLEDs showed the best performance with a turn-on voltage of 3.2 V, a luminance peak of 21357 cd m^–2^ at 13 V, a peak current efficiency of 28.8 cd A^–1^ (9.5% EQE), and a lifetime *t*_50_ of 15.7 h.

With all of these features in mind and the good performances of devices based on the mononuclear complexes with carbazole-appended cyclometalated NHC, [Pt­(Cz-C^∧^C*_im/bzim_)­(acac)] (Cz-CH^∧^C* = 1-(4-(9*H*-carbazol-9-yl)­phenyl)-3-methyl-1*H*-imidazol/benzimidazol-2-ylidene),^10^ we decided to prepare dinuclear butterfly complexes using chromophore “Pt­(Cz-C^∧^C*_im_)” in the wings and pyrazolates in the body. This approach seeks to introduce the blue-light-emitting fragment Pt­(Cz-C^∧^C*_im_) into a structure designed to hinder intermolecular interactions, thereby achieving efficient blue emitters. To this end, it is equally necessary to prevent intramolecular Pt···Pt interactions and ensure that the emission arises from monometallic fragments. To achieve this, we selected pyrazolates with nonbulky substituents in the 3 and 5 positions, such as pyrazolate (pz), 4-fluoropyrazolate (4-Fpz), and 4-trifluoromethylpyrazolate (4-CF_3_pz). As a result of our work, we report herein the synthesis and structural characterization of a novel series of platinum complexes featuring a “butterfly” core. Their photophysical profiles were systematically investigated and further elucidated through TD and TD-DFT calculations, with their applicative potential showcased through their integration into electroluminescent devices.

## Results and Discussion

### Synthesis and Characterization

Compounds [{Pt­(Cz-C^∧^C*_im_)­(μ-Rpz)}_2_] (HC^∧^C* = 1-(4-(carbazolyl) phenyl)-3-methyl-1*H*-imidazol-2-ylidene; Rpz = pyrazolate (pz) **1**; 4-fluoropyrazolate (4-Fpz) **2**; 4-trifluoromethylpyrazolate (4-CF_3_pz) **3**) were prepared following a sequential procedure, as indicated in Scheme 2 (see experimental details for **1**–**3** in the SI).

**Scheme 2:**
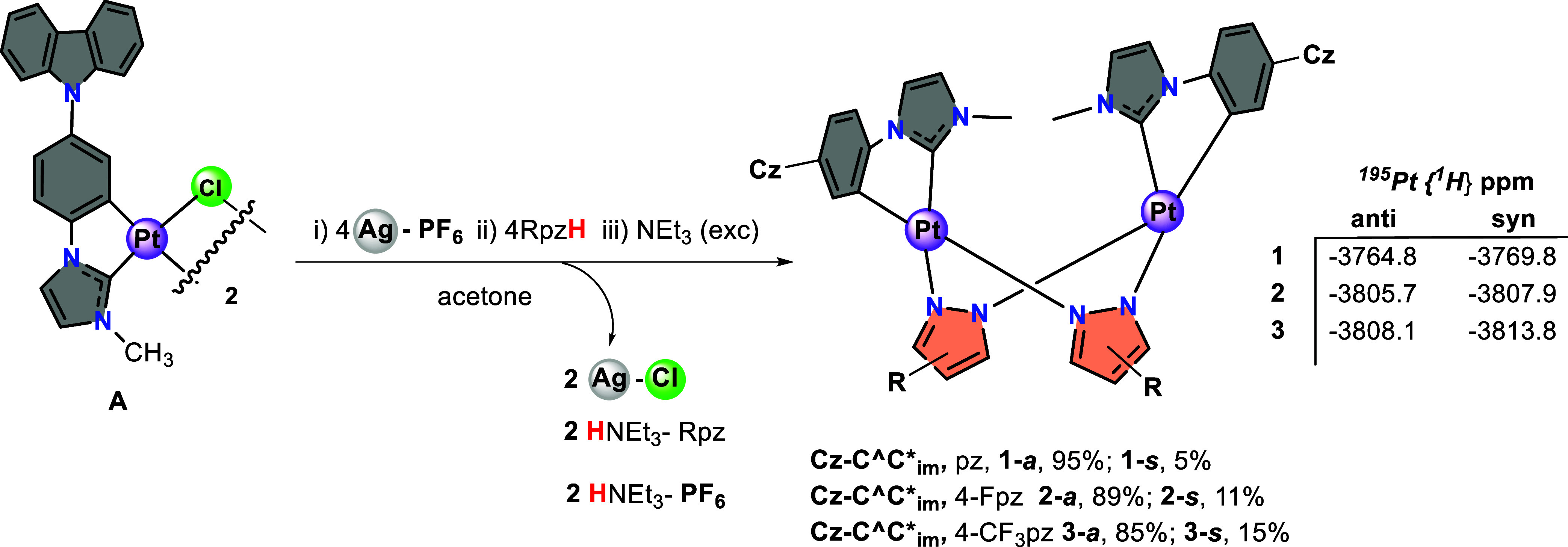
Synthetic Routes Followed for Compounds **1**–**3** (*a = anti*, *s = syn*)

All of the complexes were obtained as a mixture of *anti/syn* isomers with respect to the relative orientation of the cyclometalated C^∧^C* groups, with the *anti* isomer being the major product, as can be seen in the ^1^H and ^195^Pt­{^1^H} NMR spectra of **1**–**3** (Figures S1–S3). The values of δ^195^Pt­{^1^H} NMR show that the ^195^Pt resonances of **2** and **3** appear slightly high-field shifted with respect to that of **1**, in both the *anti* and *syn* isomers. Therefore, the presence of electron-withdrawing groups (F, CF_3_) on the pyrazolate bridging groups does not appear to decrease the electron density at the platinum center.

The crystal structures of compounds **1-a**, **2-a**, and **3-a** are depicted in Figure 1. They show a spread butterfly-like structure with intermetallic separation in the range observed in analogous compounds with different cyclometalated NHC carbenes,^23,25,28^ which are large enough to ensure the absence of a metal–metal bond,^29–32^ but not to preclude some degree of intermetallic interaction.^29,33^ A full description of these X-ray structures is included in the Supporting Information (Table S2 and Figures S4 and S5). The absence of significant π–π intermolecular interactions can be evidenced from their packing arrangement, as shown in Figure S5.

**Figure 1:**
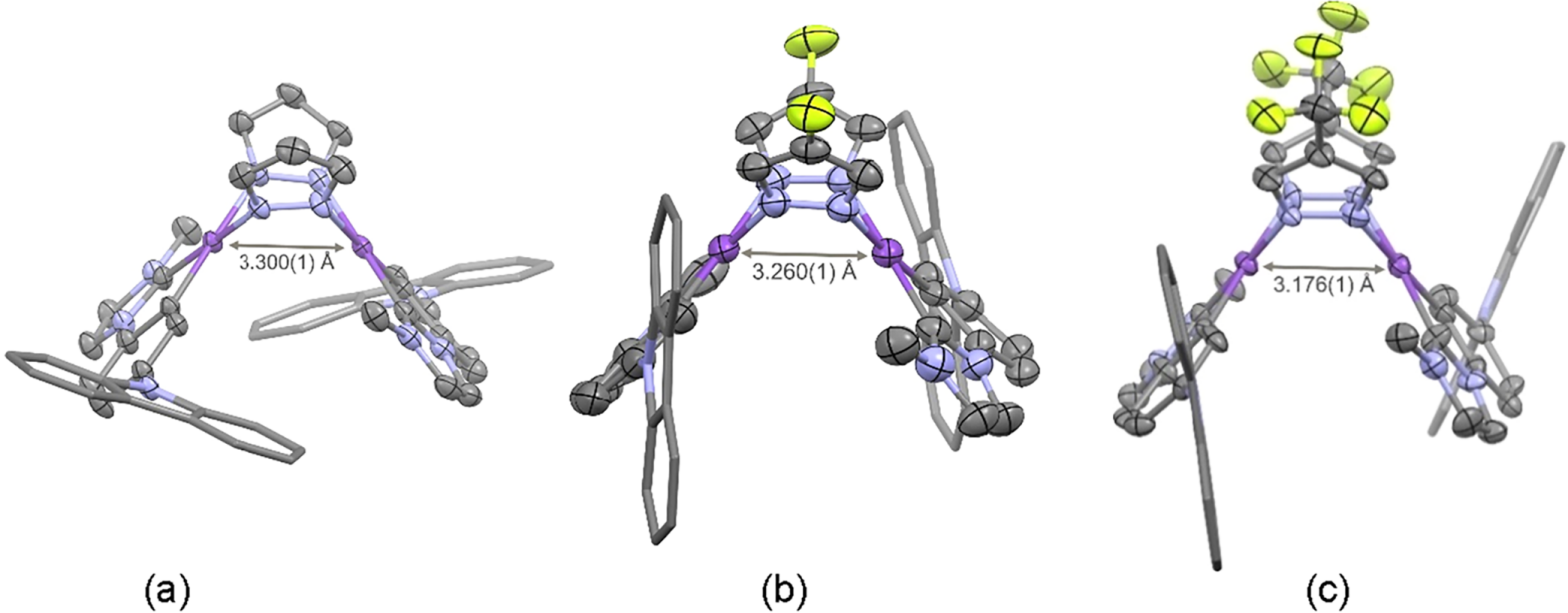
Molecular structures of **1**(a), **2**(b), and **3**(c). Solvent molecules and hydrogen atoms have been omitted for clarity.

Thermogravimetric analysis (TGA) recorded in a N_2_ atmosphere reveals that complexes **1**–**3** suffer significant weight loss at temperatures above 350 °C (387 °C, **1**; 403 °C, **2**; 355 °C **3**; see Figure S6). The high thermal stability of these complexes makes them suitable as phosphorescent dopants for OLEDs processed using high-vacuum techniques.

### Computational Results

To shed light on the nature of low-energy UV–vis absorption bands and on the emission properties of **1**–**3**, we performed density functional theory (DFT) and time-dependent DFT (TD-DFT) calculations for complexes **2-a** and **3-a** in THF solution (see computational details in the SI). The optimized geometries of **2-a** and **3-a** in their ground state (GS, S_0_) display Pt–Pt distances (3.357 and 3.378 Å, respectively) slightly longer than those observed in the X-ray structures (Figure S7). Accordingly, due to long intermetallic distance, these structures are characterized by a small Pt–Pt Mayer bond order (0.049, **2**-**a**; 0.035, **3-a**). By comparing these computed values with those for complexes [{Pt­(EtO_2_C-C^∧^C*)­(μ-Rpz)}_2_] (HC^∧^C* = 1-(4-(ethoxy-carbonyl)­phenyl)-3-methyl-1*H*-imidazol-2-ylidene; Rpz = pz **Ref1**, 4-Mepz **Ref2**),^25^ we observed that they are similar to those found for the butterfly spread calculated conformers (d_Pt–Pt_ = 3.20 Å, **Ref1**; 3.22 Å, **Ref2**; Mayer BO: 0.11, **Ref1**; 0.111, **Ref2**) and differ from those found for the butterfly folded ones (d_Pt–Pt_ = 2.97 Å, **Ref1**; 2.98 Å, **Ref2**; Mayer BO: 0.228, **Ref1**; 0.233, **Ref2**).^25^ Thus, it can be inferred that these complexes exhibit a butterfly spread conformation. We also optimized the geometries of **2-a** and **3-a** in their lowest triplet excited state (T_1_) using unrestricted UDFT calculations (Figure S7). The T_1_-optimized geometries display Pt–Pt distances (3.343 Å, **2-a**; 3.365, Å **3-a**) and Mayer BO (0.034, **2-a**; 0.018, **3-a**) rather similar to those found in the GS-optimized geometries. These values agree with the calculated spin-density distribution plots (Figure 2), where the electron density is mainly located in the C^∧^C*_im_ ligand and, to a minor extent, at the Pt center, with no participation of the pyrazolate ligands. This indicates a mixed ^3^IL/^3^MLCT character for T_1_, ruling out a putative ^3^MMLCT character, which is often found when the intermetallic distances are shorter than 3.0 Å.

**Figure 2:**
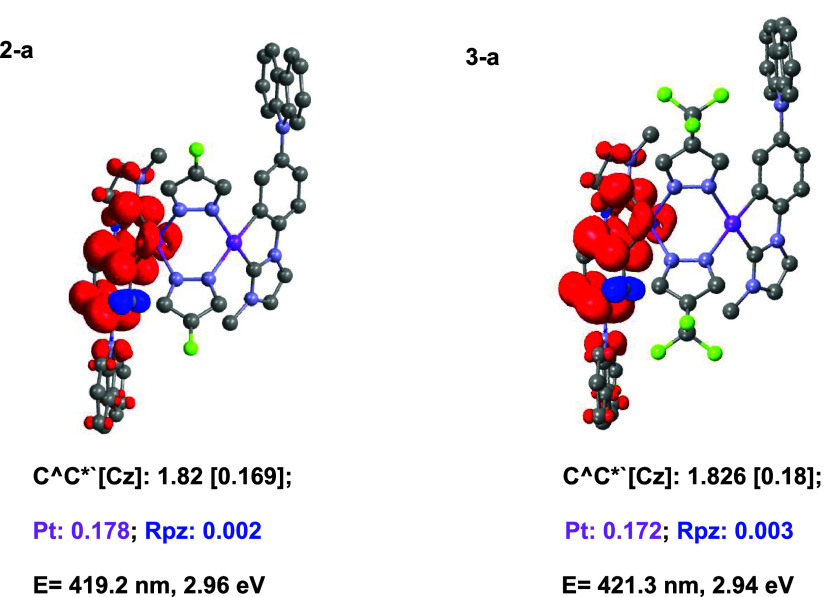
Spin-density distributions for the optimized T_1_ states of **2-a** and **3-a** calculated in THF by UDFT. Vertical emission energies for T_1_ were calculated by TD-DFT.

The absorption and emission properties were also investigated by TD-DFT calculations (Photophysical and Electrochemical Properties section). The energy and composition of the frontier molecular orbitals (Figure S8 and Table S3) and the energy (λ_calc_, nm) and nature of the lower singlet and triplet excited states are detailed in the SI (Table S4). There are many similarities between the two complexes, **2-a** and **3-a**. For both, the highest occupied molecular orbitals, HOMO and H_–1_, are degenerated, and are mostly built from Cz-C^∧^C*_im_ orbitals, especially from the Cz fragment (73%); H_–2_ is mainly built up by the d_z_^2^ orbitals of the two Pt atoms (≈75%); in contrast, the lowest unoccupied molecular orbital, LUMO, is from the orbitals of Pt (≈ 39%) and C^∧^C*_im_ (60%). The nature of the lower-energy absorptions and emissions is also very similar, as discussed in the next section.

### Photophysical and Electrochemical Properties

The UV–vis spectra of complexes **1**–**3** in 5·10^–5^ M solutions in THF display very similar features (Figure 3 and Table S5 and Figure S9 in the SI). They show intense absorption bands at λ < 300 nm, attributed to ^1^IL transitions localized on the cyclometalated NHC ligand (Cz-C^∧^C*_im_) and less intense absorption bands in the range 320–350 nm tailing up to 400 nm. The experimental low-energy absorption bands of **2** and **3** match the lowest-lying TD-DFT singlet vertical excitation energies for **2-a** and **3-a**, depicted as vertical bars in Figure 3. Specifically, the S_0_ → S_1_ (*f* = 0.0491) and S_0_ → S_3_ (*f* = 0.2721) transitions contribute mainly to these bands in **2-a**, while the S_0_ → S_2_ (*f* = 0.0974) and S_0_ → S_3_ (*f* = 0.2687) transitions do so in **3-a**. To visualize these results, the natural transition orbitals (NTOs) of these transitions were calculated and are illustrated in Figure 3. Analysis of the NTOs reveals a mixed character^1^ILCT/^1^LMCT [π­(Cz) → π­(C^∧^C*)]/[π­(Cz) → d­(Pt)] for S_1_/S_2_ in **2-a**/**3-a** and ^1^MLCT [d_z_^2^(Pt) → π­(C^∧^C*)] character for S_3_ in both complexes.

**Figure 3:**
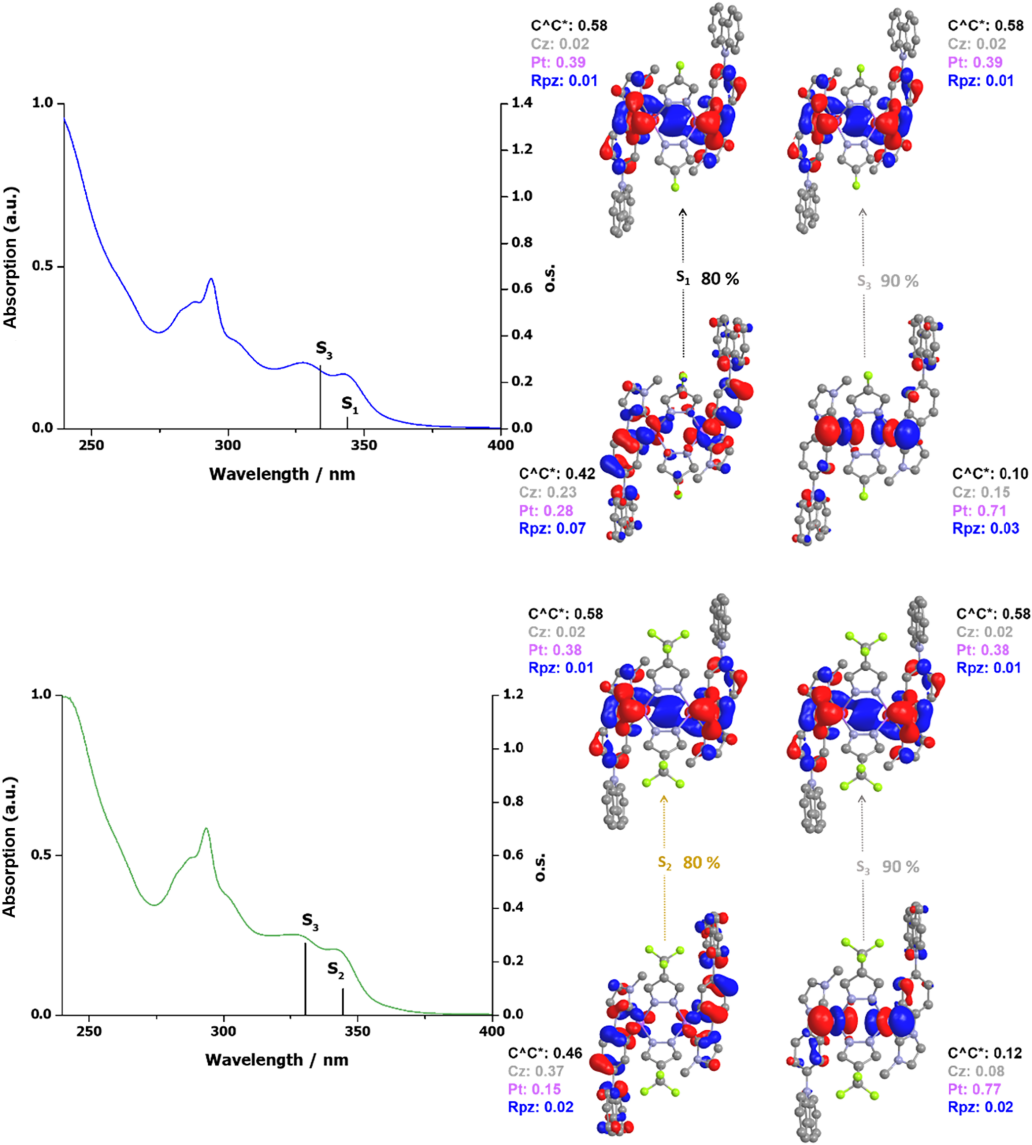
UV–vis spectra of **2** (top) and **3** (bottom) in THF, with calculated transitions (bars) and plots of the calculated NTOs (isoval. 0.03) for the main contributing transitions.

These absorptions appear clearly shifted to the blue with respect to **Ref1** and **Ref2**.^25^ If we focus on the butterfly compounds **1** (λ_abs_, nm: 328, 343 tail to 375) and **Ref1** (λ_abs_, nm: 335, 354 tail to 425), both bearing pz as the bridging ligand, the data reveal that the Cz-C^∧^C* wings raise the energy of the excited states more than the EtO_2_C-C^∧^C* wings. This observation can be attributed to the destabilization of the LUMO, localized on the C^∧^C* fragment, arising from the electron-donating character of the Cz moiety.

In line with the absorption spectra, cyclic voltammetry (CV) of **1**–**3**, carried out in CH_2_Cl_2_ as the solvent and using the Fc^+^/Fc redox couple as the internal standard, shows similar electrochemical behavior for these compounds. The obtained voltammograms are depicted in Figures 4 and S10 in the SI, and the relevant data is summarized in Table 1.

**Figure 4:**
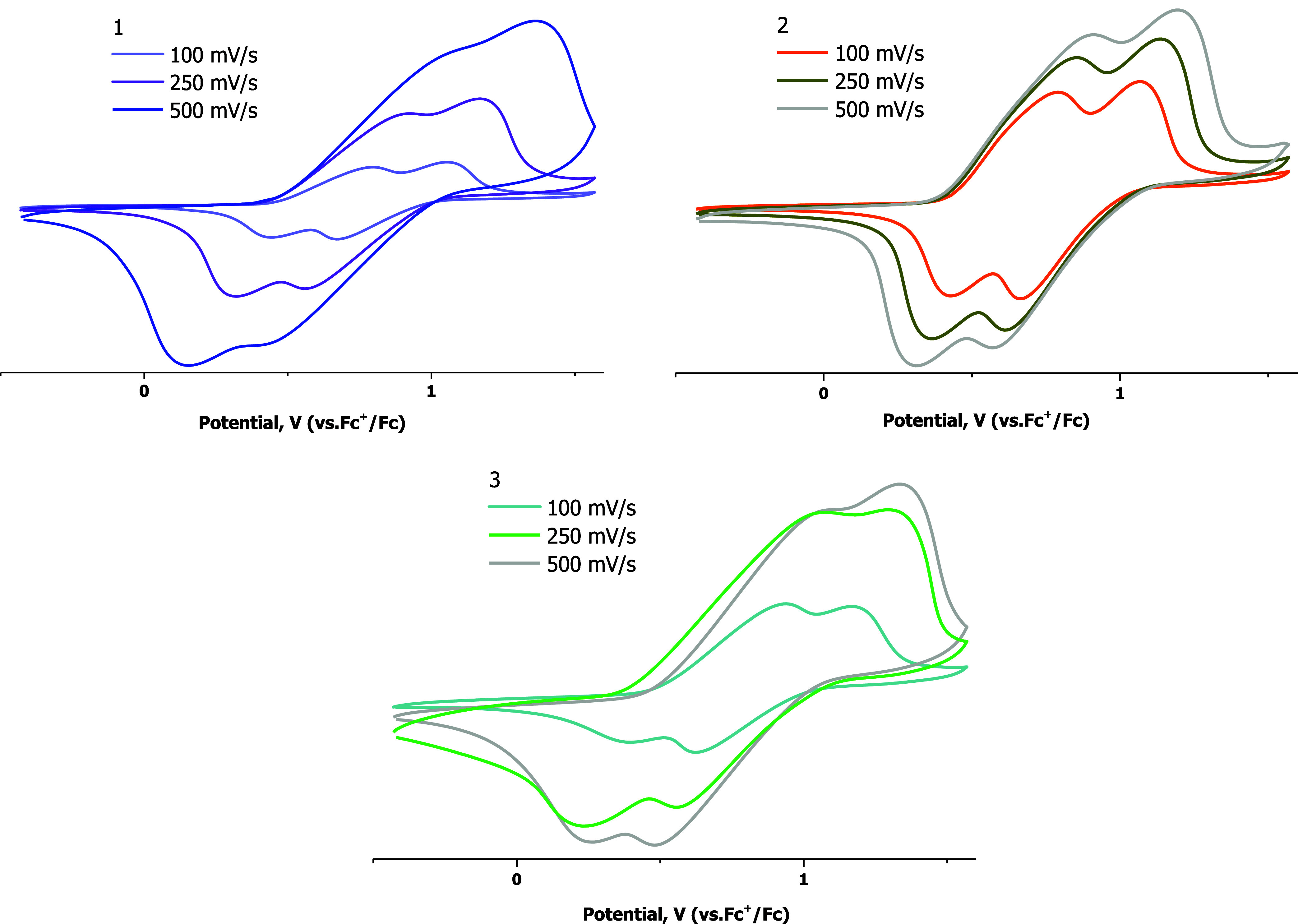
Cyclic voltammograms of complexes **1**–**3** in CH_2_Cl_2_ at 100 mV s^–1^.

**Table 1:** Electrochemical Data and HOMO/LUMO Energy Estimation^c^

com.	*E*_onset ox1_ ^a^	*E*_HOMO_ ^b^	*E*_LUMO_ ^b^	*E*_g_ ^b^
**1**	0.45	–5.55	–2.11	3.44
**2**	0.43	–5.53	–2.09	3.44
**3**	0.49	–5.59	–2.12	3.47

a V

b eV

c 0.5 M (Bu_4_N)­PF_6_ in CH_2_Cl_2_, 100 mV s^–1^ scan rate, measured against Ag/AgCl using the Fc^+^/Fc redox couple as a reference.

Compounds **1**–**3** exhibit well-defined anodic and cathodic waves with substantially broadened peak profiles and pronounced scan-rate-dependent peak separations. The marked increase in Δ*E*_p_ while increasing the speed rate is indicative of a quasi-reversible redox process. The HOMO energy values were calculated in the Fermi scale,^34^ from the first oxidation wave as *E*_HOMO_ (eV) = −(*E*_onset_ _ox1_ + 5.1). The LUMO energy values were calculated from those of the HOMO and *E*_g_ (*E*_g_= 1240/λ­(nm)) obtained from the UV–vis spectra as *E*_LUMO_ (eV) = *E*_HOMO_ + *E*_g_ eV.^35^ Considering the composition and calculated energies of the highest occupied molecular orbitals in **2-a** and **3-a** (*E*_HOMO/H‑1_ = −5.7 eV), the first oxidation process can be assumed to occur on Cz of the Cz-C^∧^C*_im_ ligand. The similar shape and *E*_ox1_ values observed for the oxidation of complexes **1**–**3** and [Pt­(Cz-C^∧^C*_im_)­(acac)][Bibr ref10] reflect the key role of the pendant Cz in the electrochemical behavior of Pt­(II) complexes bearing the cyclometalated Cz-C^∧^C*_im_ group.

The photoluminescence properties of all of the complexes were examined in 5 wt % doped poly­(methyl)­methacrylate (PMMA) films (Table 2 and Figure 5). Complexes **1**–**3** exhibit intense phosphorescence in the CIE’s blue corner upon excitation at 350 nm, being **1** a “pure-blue” (CIE_*x*+*y*_ < 0.3)^36^ emissive complex. This was expected since the HOMO–LUMO gap (*E*_g_) is greater than 3.4 eV (Table 1), above the minimum required value (2.8 eV) for blue emitters.^37^ The emission spectra of **1**–**3** are independent of the excitation wavelength and doping concentration (Figure S11 for **1** or **2**) and almost match one another, indicating a negligible effect of the bridging pyrazolate groups on emission. The spectra exhibit a vibronic structure (peak spacing: 920–1250 cm^–1^) similar to that observed in the mononuclear complexes [Pt­(Cz-C^∧^C*_im_)­(P^∧^O)] (P^∧^O: 2-diphenylphosphinobenzoate, 2-diphenylphosphinophenolate), along with emission lifetimes in the microsecond range.^10^ Consistent with these observations, spin-density distributions calculated for the T_1_-optimized states of **2-a** and **3-a** in THF solution (Figure 2) indicate a predominant ^3^IL­[C^∧^C*] character of the T_1_ state, with some contribution from ^3^MLCT. The small contribution of metal orbitals to the emissive state, together with structural features of the platinum butterfly complexes that restrict intramolecular motion and thus reduce *k*_nr_ values, likely accounts for the high PLQYs (up to 0.99 under an Ar atmosphere) observed for these complexes (see Table 2).

**Figure 5:**
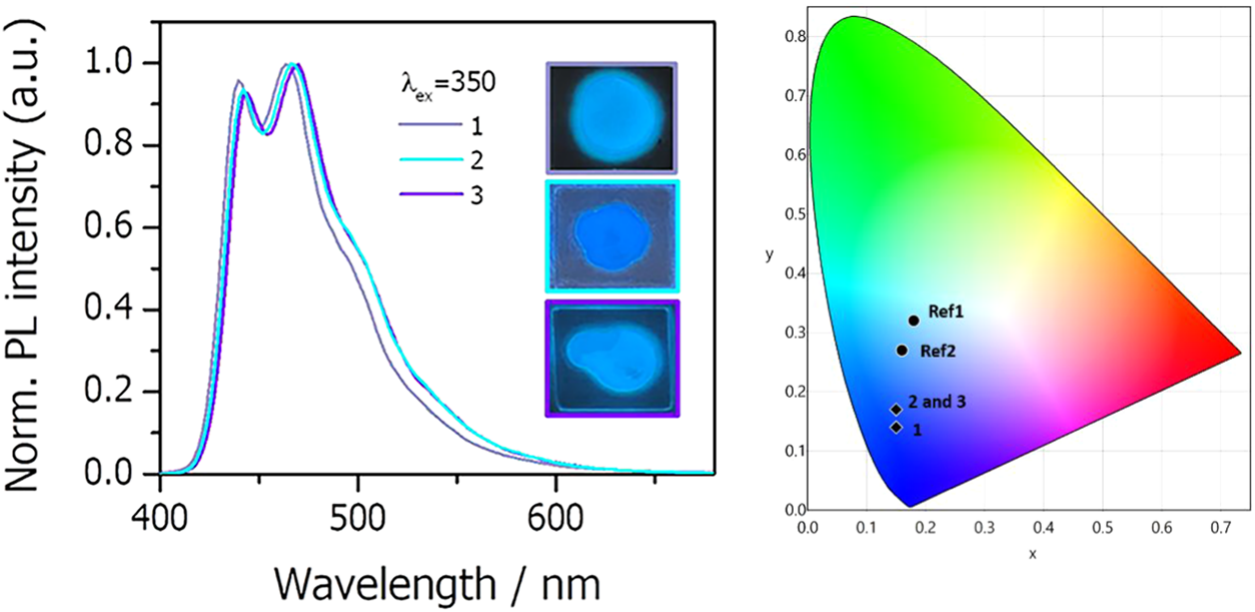
Emission spectra of complexes **1–3** in PMMA films at 5 wt % (left) and CIE coordinates of the corresponding emission (right).

**Table 2:** Photophysical Data for **1**–**3** in PMMA at 5 wt %^d^

comp.	media	λ_exc_ (nm)	λ_em_ (nm)	CIE (*x*, *y*)	*τ* (μs)	QY_PL_	*k*_*r*_	*k*_*nr*_	ΔES-T^b^
**1**	PMMA^a^	350	440, 464_max_, 494_sh_	0.15, 0.14	6.3	0.99	1.6·10^5^	0.2·10^4^	0.745
	PMMA^c^	350	440, 464_max_, 494_sh_	0.15, 0.14	4.3	0.99	2.3·10^5^	0.2·10^4^	
	2-MeTHF								
	10^–5^M, 77K	350	432_max_, 460, 486 tail to 600	0.15, 0.11	9.7				
**2**	PMMA^a^	350	442, 466_max_, 496_sh_	0.15, 0.17	7.6	0.99	1.3·10^5^	0.1·10^4^	0.738
	PMMA^c^	350	442, 466_max_, 496_sh_	0.15, 0.17	5.6	0.90	1.6·10^5^	1.8·10^4^	
**3**	PMMA^a^	350	444, 470_max_, 498_sh_	0.15, 0.17	9.5	0.93	0.9·10^5^	0.7·10^4^	0.790
	PMMA^c^	350	444, 470_max_, 498_sh_	0.15, 0.17	6.8	0.70	1.0·10^5^	4.4·10^4^	
**Ref1**	PMMA^c^	350	483_max_, 517_sh_, 567_sh_	0.18, 0.32		0.72			
	PMMA^c^	390	483_max_, 517_sh_, 567_sh_	0.18, 0.32	3.7	0.20	5.4·10^4^	2.2·10^5^	
**Ref2**	PMMA^c^	370	473, 492_max_, 536_sh_	0.16, 0.27		0.83			
	PMMA^c^	390	469, 485_max_, 524_sh_	0.16, 0.29	3.5	0.54	1.5·10^5^	1.3·10^5^	

a Under Ar.

b Calculated from excitation and emission spectra.

c In air; κ_r_ = QY/τ; κ_nr_ = (1-QY)/τ.

d PMMA film at 5 wt %.

Consistent with absorption profiles, the emission maxima of **1**–**3** in 5 wt % PMMA films are clearly blue-shifted relative to those of **Ref1** and **Ref2**. This trend is quantitatively reflected in the CIE chromaticity coordinates; notably, complex **1** exhibits a pure-blue emission (CIE_*x*+*y*_ < 0.3). Also, it is worth noting their high PLQYs despite their longer excited-state lifetimes. This improvement is primarily ascribed to a tenfold increase in the radiative rate constant (*k*_r_), accompanied by a current-order-of-magnitude reduction in the nonradiative rate constant (*k*_nr_). Given the potential for “butterfly” flapping motions to facilitate nonradiative processes, we hypothesize that the bulky Cz substituent introduces steric hindrance, likely suppressing such a nonradiative deactivating pathway and rendering highly efficient phosphorescent emitters. Notably, complexes **1**–**3** with nonbulky 3,5-disubstituted pyrazolate bridging ligands are among the stronger blue phosphorescent complexes reported to date for Pt butterfly complexes,^18^ establishing a new benchmark for blue-emitting phosphors within the [{Pt­(RC^∧^C*)­(R’pz)}_2_] structural class.

### Electroluminescence

Given their interesting optical properties, including their high QY_PL_ (Table 2) and the potential for deep-blue emission, PhOLEDs were prepared using these complexes as active materials. The performance summary of the devices prepared in this work is provided in Table S6. Device fabrication details for the different stacks are provided in the Experimental Section of the Supporting Information.

The starting selected device architecture was ITO (150 nm)/PEDOT:PSS (35 nm)/TAPC (40 nm)/mCP (10 nm)/mCPCN:10 wt % complex **1** (30 nm)/PO-T2T (60 nm)/Ba (3 nm)/Ag (100 nm), where PEDOT:PSS is poly­(ethylenedioxythiophene):poly­(styrenesulfonate), TAPC is 1,1-bis­[(di-4-tolylamino)­phenyl]­cyclohexane, mCP is 1,3-bis­(*N*-carbazolyl)­benzene, mCPCN is 9-(3-(9*H*-carbazol-9-yl)­phenyl)-9*H*-carbazole-3-carbonitrile, and PO-T2T is 2,4,6-tris­[3-(diphenylphosphinyl)­phenyl]-1,3,5-triazine, and these compounds were used as the hole-injection, hole-transport, hole-transport, host, and electron-transport materials, respectively. mCPCN was selected as the host due to its good carrier-transport properties and general compatibility with wide-band-gap blue emitters^10^ (see the energy-level diagram, Figure S12a). All layers were thermally evaporated, except for PEDOT:PSS, which was processed from solution.

Devices showed cyan electroluminescence (EL) with a maximum peak at 510 nm (Figure S13a) and CIE coordinates of (0.14, 0.46) (Figure S13b). The EL can be described as a combination of three peaks: the aforementioned maximum EL (510 nm), and two shoulders at around 450 and 490 nm, which are analogous to those observed in the PL spectrum. The current density–voltage–luminance (*J*–*V*–*L*) characteristics and the external quantum efficiency (EQE) versus luminance are presented in Figure S13c,d. The devices showed a maximum luminance of 949.2 cd m^–2^ and a maximum EQE of 0.40%.

Given the low EQE values obtained, we decided to switch to solution processing while keeping the same EML. In this case, the selected device stack was ITO (150 nm)/PEDOT:PSS (35 nm)/mCPCN:10 wt % complex **1** (30 nm)/PO-T2T (60 nm)/Ba (3 nm)/Ag (100 nm) (Figure S12b). The resulting devices showed an improvement in both the maximum luminance (2219.9 cd m^–2^) and the EQE (2.50%) (Figure S14c,d). Moreover, their EL was blue-shifted, with the same three contributions, and the maximum peak was at 489 nm (Figure S14a). This was also reflected in the chromaticity diagram coordinates (0.13, 0.34) (Figure S14b). Despite this, the devices did not reach the pure-blue emission observed in the PL spectra (CIE_*x*+*y*_ > 0.3).^36^

A different route to achieving deep-blue emission is to use the Pt­(II) complex as a sensitizer in phosphorescent sensitized thermally assisted delayed fluorescent systems (PS-TADF OLEDs).^38^ In these systems, the phosphor complex acts as an assistant dopant that transfers its electrically generated energy (by Förster energy transfer) to the excited state of the terminal emitter, usually a multiresonant TADF molecule (MR-TADF), used due to their narrow emission and high EQE.^2^ We tested this by preparing devices with the stack architecture ITO (150 nm)/PEDOT:PSS (35 nm)/mCPCN:10 wt % Pt_2_ complex (**1**–**3**):1 wt % ν-DABNA (30 nm)/PO-T2T (60 nm)/Ba (3 nm)/Ag (100 nm), where ν-DABNA is *N*7,*N*7,*N*13,*N*13,5,9,11,15-octaphenyl-5,9,11,15-tetrahydro-5,9,11,15-tetraaza-19b,20b-diboradinaphtho­[3,2,1-de:1′,2′,3′-jk]­pentacene-7,13-diamine and was used as the MR-TADF terminal emitter (Figure S12c). υ-DABNA was selected as the terminal emitter because its absorption band overlapped the photoluminescence emission of the Pt-complex sensitizer, ensuring efficient energy transfer (Figure S15). The three complexes (**1**–**3**) were tested by this approach. A sample without a sensitizer was also prepared as a reference. In all cases, the EL shows a sharp and narrow peak at 476 nm (Figure S16a), corresponding to the EL emission of the υ-DABNA terminal emitter (CIE_*x*,*y*_ = 0.11, 0.19), with a clear blue emission on the chromaticity diagram (Figure S16b). By comparing the *J–V–L* characteristics, we observed an improvement in the performance metrics when incorporating the Pt­(II) complex as a sensitizer, with the best devices (complex **2**) achieving a luminance of 4646.1 cd m^–2^ and an EQE of 6.71% (reference υ-DABNA device: 4646.1 cd m^–2^ and 4.25%) (Figure S16c,d and Table S6).

Despite this, the EQE of the different approaches carried out in this work remains rather modest, with a maximum EQE of 2.50% when the Pt­(II) complex acts as an emitter. The origin of this low EQE might reside in the proximity of the triplet energy levels of both the host (mCPCN) and the complexes. Indeed, mCPCN has a triplet energy of 3.03 eV,^39^ which is very close to the calculated triplet energy state of the complexes, 2.96 and 2.94 eV for complexes **2** and **3**, respectively (see Figure 2). These close energies can lead to quenching of the excitons from the complex to the host, where the triplet energy is transferred back to the host. Indeed, effective triplet confinement in the emitter requires triplet energy differences larger than 0.2 eV.^40^ To check this, we selected TSPO1 (diphenyl­[4-(triphenylsilyl)­phenyl]­phosphine oxide) as a host, which is known to have a very high triplet state energy (3.36 eV),^41^ and prepared devices with the following architecture: ITO (150 nm)/PEDOT:PSS (35 nm)/TAPC (40 nm)/TCTA (10 nm)/TSPO1:10 wt % di-Pt complex (**1**–**3**) (30 nm)/PO-T2T (60 nm)/Ba (3 nm)/Ag (100 nm), where TCTA is tris­(4-carbazoyl-9-ylphenyl)­amine (Figure S12d). The PEDOT:PSS layer was processed from solution, while the rest of the layers were thermally evaporated.

The EL of the devices exhibited cyan emission with broad peaks, showing maximum intensities at 503 (complex **1**) and 509 nm (complex **2** and **3**), with a secondary peak at 446 nm and shoulder at 485 nm (more pronounced in complex **1**) corresponding to the two main peaks observed in the PL spectra (Figure 6a). This resulted in CIE coordinates in the chromaticity diagram of (0.14, 0.46), (0.15, 0.37), and (0.17, 0.44) for complexes **1**, **2**, and **3**, respectively (Figure 6b). The differences in the relative contributions of the EL peaks, compared to the measured PL in PMMA films, can be ascribed to wavelength-dependent light outcoupling and, to a lesser extent, to preferential population of lower-energy excited states, as supported by comparisons between the PL of the full device stack, the PL in PMMA films, and the EL spectra (Figure S17).^42,43^ Additionally, the slightly red shift compared to devices using mCPCN as a host could be attributed to differences in dipole moment between the two host materials.^44^ The *J*–*V*–*L* characteristics were also analyzed (Figure 6c,d). The devices showed luminances of 2357.7, 1093.9, and 1261.8 cd m^–2^ and EQEs of 4.26%, 3.60%, and 4.02% for complexes **1**, **2**, and **3**, respectively. Interestingly, when comparing these devices with those using mCPCN as a host, we observed a sharp increase in the efficiency, a clear indication that the energy of the triplet state of the host influences the performance of these devices. However, this value is still lower than expected when compared to the best state-of-the-art Pt­(II) PhOLEDs,^45^ indicating additional device-level losses. The relatively high turn-on voltage is consistent with injection/transport limitations that can induce imperfect charge balance and electrically quenching.

**Figure 6:**
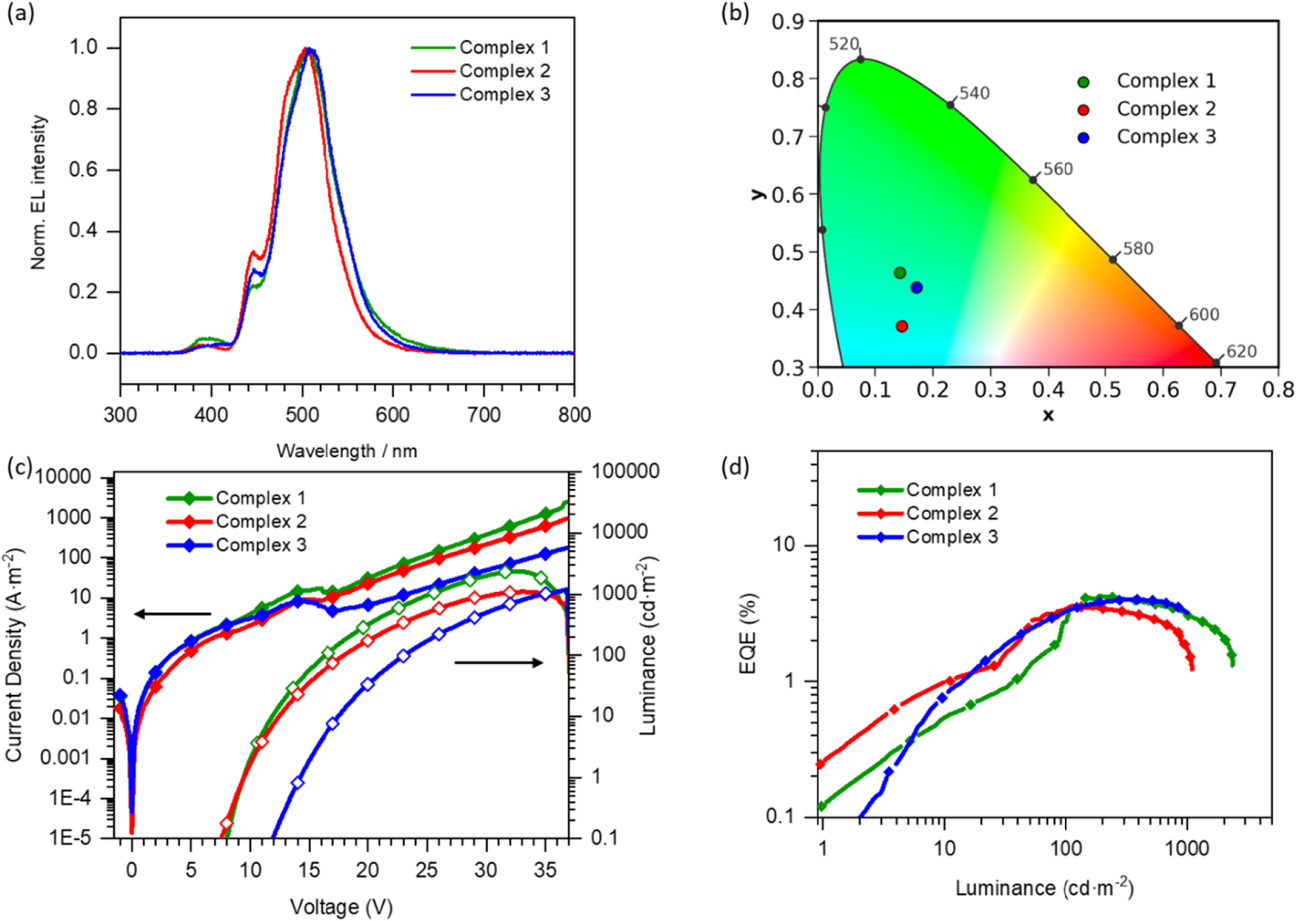
Thermally evaporated PhOLEDs using TSPO1 as the host and complexes **1*–*3** as guests. (a) Electroluminescence spectra, (b) color points in the chromaticity diagram, (c) *J*–*V*–*L* characteristics, and (d) EQE versus luminance plot.

## Conclusions

Compounds [{Pt­(Cz-C^∧^C*_im_)­(μ-Rpz)}_2_] (Rpz = pz, **1**; 4-Fpz, **2**; 4-CF_3_pz, **3**) were obtained as the major *anti* isomer from the precursor [{Pt­(Cz-C^∧^C*_im_)­(μ-Cl)}_2_]. They exhibit a butterfly spread conformation with long intermetallic distances (d_Pt···Pt_ ≈ 3.2 Å), as expected given the low bulkiness of the selected pyrazolate bridging ligands. The presence of the pendant Cz in the cyclometalated Cz-C^∧^C*_im_ group plays a key role in both photophysical and electrochemical properties, as it influences either the composition or the energies of the frontier orbitals (HOMO, H_–1_, and LUMO).

Complexes **1**–**3**, featuring nonbulky 3,5-disubstituted pyrazolate bridging ligands, are among the stronger blue phosphorescent Pt butterfly complexes reported to date. In 5 wt % PMMA films, their emissions—originating from ^3^IL/MLCT states—exhibit PLQYs of up to 0.99 and fall within the CIE_*x*,*y*_ blue corner, with complex **1** achieving “pure-blue” emission (CIE_*x*+*y*_ < 0.3). The comparison of the PLQY of complex **1** with that of its **Ref1** analogue lead us to hypothesize that the bulky Cz substituent introduces steric hindrance that hinders the deactivating butterfly flapping motion and makes blue emitters more efficient. Complexes **1**–**3** were used as emitters in PhOLEDs, achieving a maximum EQE of 4.26% and luminance of 2357.7 cd m^–2^, with cyan electroluminescence (CIE_*x*,*y*_ = 0.14, 0.46) (complex **1**). Additionally, they were used as sensitizers in PS-TADF OLEDs, yielding blue devices with EL corresponding to the υ-DABNA terminal emitter. The best performance was obtained with complex **2**, which exhibited CIE_*x*,*y*_ = 0.11, 0.19 and an EQE of 6.71%.

## Experimental Section

### General Procedures, Materials, and Instrumentation

Compound [{Pt­(Cz-C^∧^C*_im_)­Cl­(NCCH_3_)] **(**HC^∧^C*_im_ = 1-(4-(carbazolyl)­phenyl)-3-methyl-1*H*-imidazol-2-ylidene) was prepared as described elsewhere.^46^ Pyrazole was purchased from Alfa Aesar; 4-fluoropyrazole and 4-trifluoromethylpyrazole were from FluoroChem and used as received. Aqueous poly­(3,4-ethylenedioxythiophene) polystyrene sulfonate (PEDOT:PSS) dispersion was used as a hole-injection layer and was purchased from Clevios (P VP AI 4083). Organic hole-transport materials (Di-[4-(*N*,*N-*di-*p*-tolyl-amino)-phenyl]­cyclohexane (TAPC, sublimed >99.5%), 1,3-bis­(*N*-carbazolyl)­benzene (mCP, sublimed >99.5%), and 4,4′,4-tris­(carbazol-9-yl)­triphenylamine (TCTA, sublimed >99.5%)), hosts (9-(3-(9*H*-carbazol-9-yl)­phenyl)-9H-carbazole-3-carbonitrile (mCPCN, sublimed >99%) and diphenyl-4-triphenylsilylphenyl-phosphine oxide (TSPO1, sublimed >99%)), terminal emitter (5*H*,9*H*,11*H*,15*H*-[1,4] benzazaborino [2,3,4-kl]­[1,4] benzazaborino [4′,3′,2′:4,5]­[1,4] benzazaborino [3,2-b] phenazaborine-7,13-diamine, *N*_7_,*N*_7_,*N*_13_,*N*_13_,5,9,11,15-octaphenyl (υ-DABNA, sublimed >98%)), and the electron-transport material (2,4,6-tris­[3-(diphenylphosphinyl)­phenyl]-1,3,5-triazine (PO-T2T, sublimed >99%)) were obtained from Lumtec. All reagents used to make the OLEDs were stored in N_2_ until use without any further purification, except for PEDOT:PSS.

Mass spectral analyses were performed with Microflex MALDI-TOF Bruker or an Autoflex III MALDI-TOF Bruker instruments. C, H, and N analyses were carried out using a PerkinElmer 2400 CHNS analyzer or a Thermo Flash 1112. ^1^H, ^13^C APT, ^19^F, and ^195^Pt NMR spectra were recorded on Bruker NEO 400 and 500 MHz instruments using the standard references: SiMe_4_ for ^1^H and ^13^C, Na_2_PtCl_6_ in D_2_O for ^195^Pt, and CFCl_3_ for ^19^F. *J* values are given in Hz, and δ values are given in ppm; assignments are based on ^1^H-^1^H COSY experiments, ^1^H-^13^C HSQC and HMBC experiments. Unless otherwise indicated, all measurements were performed at r.t.

UV–visible spectra were recorded on a Unicam UV4 spectrophotometer. Steady-state photoluminescence spectra were recorded on a Jobin-Yvon Horiba Fluorolog FL-3-11 Tau 3 spectrofluorimeter. Phosphorescence lifetimes were recorded with a Fluoromax phosphorimeter accessory containing a UV xenon flash tube. Nanosecond lifetimes were recorded with a Datastation HUB-B, a nanoLED controller, and DAS6 software. Nano-LEDs emitting at 370 nm were employed for lifetime measurements. The lifetime data were fitted by using the Jobin-Yvon software package and Origin Pro 8. Solid-state quantum yields (QYs) were measured using the Hamamatsu Absolute PL Quantum Yield Measurement System C11347-11.

PL films were prepared by drop-casting solutions containing 5 wt % complex in PMMA (10^–2^ M for **1** and **2** and 2**·**10^–3^ M for **3** in CH_2_Cl_2_) onto quartz slides, followed by solvent evaporation.

Caution! The chemicals used for the synthesis and characterization are flammable (GHS02: NEt_3_, ^i^PrOH, acetone), corrosive (GHS05: AgPF_6_, NEt_3_, pzH, 4-FpzH, mCPCN), irritant (GHS07: ^i^PrOH, acetone, 4-FpzH, TCTA, PO-T2T, TSPO1, mCPCN), health hazardous, and carcinogenic (GHS06, GHS08: NEt_3_, pzH, and 4-CF_3_pzH). Therefore, all manipulations were performed on a small scale, following the procedures described in Experimental Section, and the chemicals were handled with care. In particular, all of the materials used for the fabrication of OLEDs were sublimated in a glovebox under an inert atmosphere.

### Synthesis of New Compounds

#### Synthesis of [{Pt­(Cz-C^∧^C*_im_)­(μ-pz)}_2_] (**1**)

AgPF_6_ (49.6 mg, 0.20 mmol) was added to a stirred suspension of [Pt­(Cz-C^∧^C*_im_)­(μ-Cl)]_2_ (111.1 mg, 0.10 mmol) in acetone (40 mL) in the dark at room temperature. After 4 h of reaction, pyrazole (26.7 mg, 0.39 mmol) was added to the mixture and allowed to react for 16 h in the dark. Then, the resulting suspension was filtered through Celite, and an excess of NEt_3_ was added. After 2 h of reaction, the solution was concentrated to ca. 2 mL, and ^*i*^PrOH (15 mL) was added to it. The resulting off-white solid was collected, washed with ether (3 × 5 mL), and dried in vacuum to give **1-** ***anti*** (95%) and **1-** ***syn*** (5%). Yield: 76.2 mg, 67%. Anal. Calcd for C_50_H_38_N_10_Pt_2_: C,51.37; H, 3.28; N, 11.98. Found: C, 50.99; H, 3.33; N, 11.58. ^1^H NMR data for **1-** ***anti*** (400 MHz, acetone-*d*_*6*_): δ 8.20 (d, 4H, ^3^ *J*_H‑H_ = 7.7, H_Cz_), 7.82 (d, 2H, ^3^ *J*_H‑H_ = 2.0, H_im_), 7.61 (m, 4H, H_pz_), 7.51 (d, 2H, ^3^ *J*_H‑H_ = 8.1, H_Ar_), [7.48–7.42] (m, 6H; 4H_Cz_, 2H_Ar_), 7.38 (m, 4H, H_Cz_), [7.29–7.23] (m, 6H; 4H_Cz_, 2H_im_), 7.21 (dd, 2H, ^3^ *J*_H‑H_ = 8.1, ^4^ *J*_H‑H_ = 2.1, H_Ar_), 6.11 (t, 2H, ^3^ *J*_H‑H_ = 2.0, H_pz_), 3.23 (s, 6H, Me). ^1^H NMR data for **1-** ***syn*** (400 MHz, acetone-*d*_*6*_): δ 8.06 (d, ^3^ *J*_H‑H_ = 8.3, H_Cz_), 6.33 (t, ^3^ *J*_H‑H_ = 2.0, H_pz_), 5.95 (t, ^3^ *J*_H‑H_ = 2.0, H_pz_), 3.67 (s, Me). The rest of the signals appear to overlap those of **1-** ***anti*** and cannot be not assigned. ^13^C­{^1^H}-APT NMR plus HMBC and HSQC for **1-** ***anti*** (100.6 MHz, acetone-*d*_*6*_): δ 159.4 (s, 2C, ^1^ *J*_C*‑Pt_ = 1402.8, C*), 148.7 (s, 2C, C_Ar_ Ph-Pt), 142.3 (s, 4C, C_Cz_), 139.8 (s, 2C, C_pz_), 138.5 (s, 2C, C_pz_), 135.7 (s, 2C, C_Ar_), 134.0 (s, 2C, C_Ar_), 133.7 (s, 2C, C_Ar_), 126.7 (s, 4C, C_Cz_), 123.9 (s, 4C, C_Cz_), 123.4 (s, 2C, C_im_), 122.9 (s, 2C, C_Ar_), 121.1 (s, 4C, C_Cz_), 120.4 (s, 4C, C_Cz_), 116.1 (s, 2C, C_im_), 112.5 (s, 2C, C_Ar_), 110.7 (s, 4C, C_Cz_), 106.1 (s, 2C, C_pz_), 36.6 (s, 2C, Me). ^195^Pt­{^1^H} NMR (85.6 MHz, acetone-*d*_*6*_): δ 3764.8 (s) (**1**-***anti***), 3769.8 (s) (**1** *-* **syn**). MS (MALDI+): *m*/*z* 1168.166 [M]^+^.

#### Synthesis of [{Pt­(Cz-C^∧^C*_im_)­(μ-Fpz)}_2_] (**2**)

It was prepared following the method described for **1** using AgPF_6_ (55.4 mg, 0.22 mmol), [Pt­(Cz-C^∧^C*_im_)­(μ-Cl)]_2_ (124.5 mg, 0.11 mmol), and 4-fluoropyrazole (37.7 mg, 0.44 mmol) as starting materials, giving **2-** ***anti*** (89%) and **2-** ***syn*** (11%). Yield: 98.5 mg, 75%. Anal. Calcd for C_50_H_36_F_2_N_10_Pt_2_: C,49.84; H, 3.01; N, 11.62. Found: C, 49.77; H, 2.88; N, 11.39. ^1^H NMR data for **2-** ***anti*** (400 MHz, acetone-*d*_*6*_): δ 8.20 (d, 4H, ^3^ *J*_H‑H_ = 7.8, H_Cz_), 7.82 (d, 2H, ^3^ *J*_H‑H_ = 1.8, H_im_), 7.59 (dd, 4H, ^3^ *J*_H‑F_ = 14.9, ^4^ *J*_H‑H_ = 4.0, H_pz_), 7.52 (d, 2H, ^3^ *J*_H‑H_ = 8.1, H_Ar_), [7.49–7.37] (m, 10H; 8H_Cz_, 2H_Ar_), [7.30–7.21] (m, 8H; 4H_Cz_, 2H_im_, 2H_Ar_), 3.35 (s, 6H, Me). ^1^H NMR data for **2-** ***syn*** (400 MHz, acetone-*d*_*6*_): δ 8.06 (d, ^3^ *J*_H‑H_ = 8.2, H_Cz_), 3.74 (s, Me). The rest of the signals appear to overlap those of **2-** ***anti*** and cannot be assigned. ^13^C­{^1^H}-APT NMR plus HMBC and HSQC for **2-** ***anti*** (100.6 MHz, acetone-*d*_*6*_): δ 158.0 (s, 2C, ^1^ *J*_C*‑Pt_ = 1426.5, C*), 152.5 (s, 2C, ^2^ *J*_C‑F_ = 236.7, C_pz_), 148.5 (s, 2C, C_Ar_), 142.2 (s, 4C, C_Cz_), 134.4 (s, 2C, C_Ar_), 134.1 (s, 2C, C_Ar_), 133.5 (s, 2C, C_Ar_), 126.8 (s, 4C, C_Cz_), 126.6 (s, 2C, C_pz_), 125.6 (s, 2C, ^4^ *J*_C‑F_ = 20.3, C_pz_), 123.9 (s, 4C, C_Cz_), 123.5 (s, 2C, C_im_), 123.2 (s, 2C, C_Ar_), 121.1 (s, 4C, C_Cz_), 120.5 (s, 4C, C_Cz_), 116.3 (s, 2C, C_im_), 112.7 (s, 2C, C_Ar_), 110.7 (s, 4C, C_Cz_), 36.7 (s, 2C, Me). ^19^F­{^1^H} NMR (376.5 MHz, acetone-*d*_*6*_): δ −180.82 (s, 2F, **2**-***anti***), −180.89 and −180.93 (s, **2**-***syn***). ^195^Pt­{^1^H} NMR (85.6 MHz, acetone-*d*_*6*_): δ 3805.7 (s) (**2**-***anti***), 3807.9 (s) (**2**-***syn***). MS (MALDI+): *m*/*z* 1204.191 [M]^+^.

#### Synthesis of [{Pt­(Cz-C^∧^C*_im_)­(μ-CF_3_pz)}_2_] (3)

It was prepared following the method described for **1** using AgPF_6_ (57.2 mg, 0.23 mmol), [Pt­(Cz-C^∧^C*_im_)­(μ-Cl)]_2_ (128.5 mg, 0.11 mmol), and 4-(trifluoromethyl)-1*H*-pyrazole (61.5 mg, 0.45 mmol) as starting materials, giving **3-** ***anti*** (85%) and **3-** ***syn*** (15%). Yield: 75.5 mg, 52%. Anal. Calcd for C_52_H_36_F_6_N_10_Pt_2_: C, 47.86; H, 2.78; N, 10.73. Found: C, 47.91; H, 2.55; N, 10.41. ^1^H NMR data for **3-** ***anti*** (400 MHz, acetone-*d*_*6*_): δ 8.19 (d, 4H, ^3^ *J*_H‑H_ = 7.8, H_Cz_), 8.13 (d, 4H, ^3^ *J*_H‑H_ = 4.3, H_pz_), 7.86 (d, 2H, ^3^ *J*_H‑H_ = 2.0, H_im_), 7.55 (d, 2H, ^3^ *J*_H‑H_ = 8.3, H_Ar_ Ph-Pt), 7.44 (m, 8H, H_Cz_), 7.38 (d, 2H, ^4^ *J*_H‑H_ = 2.0, H_Ar_), [7.30–7.23] (m, 8H; 4H_Cz_, 2H_im_, 2H_Ar_), 3.27 (s, 6H, Me). ^1^H NMR data for **3-** ***syn*** (400 MHz, acetone-*d*_*6*_): δ 8.03 (m, H_Cz_), 3.71 (s, Me). The rest of the signals appear overlapped those of **3-** ***anti*** and cannot be assigned. ^13^C­{^1^H}-APT NMR plus HMBC and HSQC data for **3-** ***anti*** (100.6 MHz, acetone-*d*_*6*_): δ 157.1 (s, 2C, ^1^ *J*_C*‑Pt_ = 1454.9, C*), 148.2 (s, 2C, C_Ar_), 141.9 (s, 4C, C_Cz_), 139.2 (s, 2C, C_pz_), 137.9 (s, 2C, C_pz_), 134.3 (s, 2C, C_Ar_), 133.4 (s, 2C, C_Ar_), 133.0 (s, 2C, C_Ar_), 126.8 (s, 4C, C_Cz_), 125.9 (s, 2C, *C*F_3_), 124.0 (s, 4C, C_Cz_), 123.6 (s, 2C, C_im_), 123.3 (s, 2C, C_Ar_), 121.1 (s, 4C, C_Cz_), 120.5 (s, 4C, C_Cz_), 116.3 (s, 2C, C_im_), 112.9 (s, 2C, C_Ar_), 110.7 (s, 4C, C_Cz_), 36.7 (s, 2C, Me). ^19^F­{^1^H} NMR data (376.5 MHz, acetone-*d*_*6*_): δ −55.66 (s, 6F, **3**-***anti***), −55.55 and −55.70 (s, **3-** ***syn***). ^195^Pt­{^1^H} NMR (85.6 MHz, acetone-*d*_*6*_): δ −3808.1 (s) (**3**-***anti***), −3813.8 (s) (**3**-***syn***). MS (MALDI+): *m*/*z* 1304.178 [M]^+^.

### X-ray Structure Determination—Experimental Procedures and Refinement

Crystal data and other details of the XRD analysis are listed in Table S1. Suitable crystals of **1-a**·2.475CH_2_Cl_2_, **2-a**·0.75Me_2_CO, and **3-a**·0.75C_5_H_12_, for X-ray diffraction studies, were obtained by slow diffusion of *n*-pentane into saturated solutions of **1** in CH_2_Cl_2_ and **2** and **3** in acetone. Intensity data were collected on a Bruker Apex Duo CCD diffractometer at 100 K. The radiation used in all cases was graphite monochromated Mo Kα (λ = 71.073 pm). The diffraction frames were integrated and corrected for absorption using SAINT software.^47^ The structures were solved by Patterson and Fourier methods and refined by full-matrix least-squares on *F* ^2^ using SHELXL.^48^ All non-H atoms were assigned anisotropic displacement parameters and refined without positional constraints. All H atoms were constrained to idealized geometries and assigned isotropic displacement parameters equal to 1.2 or 1.5 times the *U*_iso_ values of their attached parent atoms.

In the case of **1-a**·2.475CH_2_Cl_2_, some diffuse electron density was detected during the refinement stages. This electron density could not be modeled as discrete solvent molecules; thus, a solvent mask was calculated, and 56 electrons were found in a volume of 331 Å^3^ in 1 void per unit cell. This is consistent with the presence of 0.35 molecules of dichloromethane per formula unit, which accounts for 59 electrons per unit cell. Similarly, in the structure of **2-a**·0.75Me_2_CO, some diffuse electron density was also detected but could not be modeled as discrete moieties. For this reason, a solvent mask was calculated, and 112 electrons were found in a volume of 664 Å^3^ in 1 void per unit cell. This is consistent with the presence of 0.9 acetone molecules per asymmetric unit, which account for 115 electrons per unit cell.

Full-matrix least-squares refinement of these models against *F* ^2^ converged to the final residual indices given in Table S1. CCDC Nos. 2525360–2525362 contain the supplementary crystallographic data for **1-a**·2.475CH_2_Cl_2_, **2-a**·0.75Me_2_CO, and **3-a**·0.75C_5_H_12_

### Computational Methods

Density functional theory (DFT) geometry optimizations of the ground (S_0_) state were performed using the M06 functional^49^ with Grimme’s D3 dispersion correction,^50^ as implemented in the Gaussian 16 suite of programs.^51^ This functional was chosen in view of previous investigations in similar Pt­(II) complexes (MUE (M06) = 2.48 kcal/mol)^52^. The ECP-60-mwb pseudopotentials were chosen for platinum,^53^ and the 6-31G* ^54,55^ basis sets were used for all other atoms. The geometry optimizations of the lowest triplet state (T_1_) were performed with UDFT using the same functional and basis sets as in the S_0_ optimizations. Geometry optimizations were performed without any symmetry constraints and in THF using the polarizable continuum model (PCM).^56^ Frequency calculations were performed to determine the nature of the stationary points found. No imaginary frequencies were found for all of the optimized minima. The vertical absorption (S_1_–S_3_) and emission (T_1_) energies were calculated with time-dependent DFT (TD-DFT) using the same functional and basis sets as in the geometry optimizations. The latter results are presented in Table S4. Mulliken population analysis was carried out as implemented in the Gaussian 16 package.^51^ ChemissianLab program package was used for the analysis and graphical representation of molecular structures and orbitals and for Mayer bond order analysis. Atomic charges were calculated by using the NBO analysis option as incorporated in Gaussian 16.^51^

### Electrochemical Characterization

Cyclic voltammetry of **1**–**3** was performed in a PalmSens4 electrochemistry workstation using a 25 × 40 mm three-electrode electrochemical cell equipped with a glassy carbon working electrode (BASi, 3 mm diameter), an Ag/AgCl (3 M KCl) electrode reference (BASi), and a platinum wire auxiliary electrode (BASi, 7.5 cm x 0.5 mm diameter). All experiments were performed at 298 K under an argon atmosphere, using a degassed 5 × 10^–4^ M solution in CH_2_Cl_2_ containing 0.1 M tetrabutylammonium hexafluorophosphate as the supporting electrolyte. Measurements were conducted at scan rates of 100, 250, and 500 mV/s, and ferrocenium/ferrocene (Fc^+^/Fc) was used as the internal standard. The HOMO energies were estimated from the onset values of the oxidation waves, referenced against Fc^+^/Fc using a formal potential of 5.1 eV for the Fc^+^/Fc couple on the Fermi scale: *E*_HOMO_ = −(*E*_onset,ox_ + 5.1) eV. The LUMO energies were calculated as *E*_LUMO_ = *E*_HOMO_ + *E*_g_ eV. *E*_g_ = 1240/λ.^57–59^

### Device Fabrication and Characterization

#### Device Fabrication

Prepatterned 150 nm-thick indium tin oxide (ITO)-coated glass plates (3 × 3 cm^2^) were used as transparent conductive substrates. They were subsequently cleaned ultrasonically in tap water–detergent, deionized water, and 2-propanol baths for 5 min. After drying, substrates were placed in a UV–ozone cleaner (Jelight 42-220) for 15 min. ITO substrates were coated with a PEDOT:PSS solution at 2000 rpm for 60 s and then annealed at 150 °C for 10 min. Substrates were transferred to a vacuum chamber where TAPC (40 nm) and mCP (10 nm) or TCTA (10 nm) were deposited at evaporation rates of 0.2 Å·s^–1^ and 0.1 Å·s^–1^, respectively, controlled through quartz crystal microbalances (QCMs). For vacuum-processed devices, the active layers were deposited by coevaporation, using mCPCN or TSPO1 (hosts) and 10 wt % complex **1**, **2**, or **3** (guest), until a thickness of 30 nm was reached. For the solution-processed devices, the layers were directly prepared onto PEDOT:PSS by spin coating at 2000 rpm for 60 s; the spin-coating solutions consisted of 10 mg mL^–1^ mCPCN in chlorobenzene. For binary devices, complex **1** was added at 10 wt % relative to the host; for ternary devices, complex **1**, **2**, or **3** was incorporated at 10 wt %, together with ν-DABNA at 1 wt % relative to the host; and for the reference device, υ-DABNA was added at 1 wt % relative to the host. Finally, PO-T2T (60 nm), Ba (3 nm) and Ag (100 nm) were subsequently deposited at rates of 0.1 Å·s^–1^, 0.2 Å·s^–1^, and 0.2–0.5 Å·s^–1^, respectively, while the background pressure was around 3 × 10^–6^ bar. Shadow masks were used during metal evaporation to obtain a final active area of 8.25 mm^2^. Devices were not exposed to water vapor or O_2_ after cathode deposition nor before characterization.

#### Device Characterization

After full device fabrication, samples were introduced into a custom setup for current density–luminance–voltage (*J*–*V*–*L*) sweeps and electroluminescence (EL) characterization. For this, we employed a Keithley 2400 Source Meter and a photodiode coupled to a Keithley 6485 picoammeter or an AvaSpec-2048L detector to measure the electroluminescence spectrum. Custom LabVIEW programs were used to control the Keithleys and acquire data.

## Supplementary Material





## Notes

Crystallographic data (Table S1), spectra for characterization, full description of the X-ray structures, data and figures for absorption theoretical calculations, emission properties, and electroluminescence spectra (PDF)
Cartesian coordinates of the DFT-optimized structures for compounds **2-a** and **3-a** (ZIP) (XYZ)
