# Supplementary material for: Pure-Blue Emission at the Edge of the CIE Diagram: Carbazolyl NHC Platinum Butterfly Complexes with Near-Unity Quantum Yield and Their Application in Organic Light-Emitting Diodes
Source: Inorg Chem. 2026 May 1;65(19):10603–15. doi: 10.1021/acs.inorgchem.6c00582 (PMC13188058; doi:10.1021/acs.inorgchem.6c00582)
Supplement: Supplementary file 1 [file ic6c00582_si_001.pdf]

Supporting Information

PURE-BLUE EMISSION AT THE EDGE OF THE CIE DIAGRAM: CARBAZOLYL NHC PLATINUM BUTTERFLY COMPLEXES WITH NEAR-UNITY QUANTUM YIELD AND THEIR APPLICATION IN ORGANIC LIGHT-EMITTING DIODES

Jorge Roy,[a] Sergio Martínez-Saiz,[b] Michele Forzatti,[b] Antonio Martín,[a] Daniel Escudero, [c] Daniel Tordera,*[b] and Violeta Sicilia*[d]*

[a.] *Departamento de Química Inorgánica, Facultad de Ciencias, Instituto de Síntesis Química y Catálisis Homogénea (ISQCH), CSIC - Universidad de Zaragoza, Pedro Cerbuna 12, 50009, Zaragoza (Spain).*

[b.] *Instituto de Ciencia Molecular, Universidad de Valencia, C/Catedrático J. Beltran, 2, 46980, Paterna, Spain. E-mail: daniel.tordera@uv.es*

[c.] *Department of Chemistry, KU Leuven, Celestijnenlaan 200f - box 2404, 3001 Leuven. E-mail: daniel.escudero@kuleuven.be*

[d.] *Departamento de Química Inorgánica, Escuela de Ingeniería y Arquitectura de Zaragoza, Instituto de Síntesis Química y Catálisis Homogénea (ISQCH), CSIC - Universidad de Zaragoza, Campus Río Ebro, Edificio Torres Quevedo, 50018, Zaragoza (Spain). E-mail: sicilia@unizar.es*

CONTENTS **Page**

1. Experimental Section: Table S1. Crystallographic data S3

2. Results and Discussion:

2.1. Spectra for characterization (Figures S1 – S3) S4

2.2. Full description of X-ray Molecular structures (Tables S2; Figures S4, S5) S15

2.3. TGA analysis (Figure S6) S18

2.4 Computational results (Tables S3, S4; Figures S7, S8) S19

2.5 Photophysical and electrochemical properties (Table S5; Figures S9-S11) S22

2.6. Electroluminescence S24

3. References S28

1. Experimental Section

Table S1: Crystallographic data

	**1-*a***·2.475CH_2Cl_2	**2-*a***·0.75Me_2CO	**3-*a***·0.75C_5H_{12}
Empirical formula	$C_{50}H_{38}N_{10}Pt_2$·2.475CH_2Cl_2	$C_{50}H_{36}F_2N_{10}Pt_2$·0.75$Me_2CO$	$C_{52}H_{36}F_6N_{10}Pt_2$·0.75$C_5H_{12}$
Formula weight	1379.289	1300.896	1359.19
Crystal system	Triclinic	Monoclinic	Monoclinic
Space group	*P*-1	$P2_1$/c	$P2_1$/c
a (Å)	10.3664(12)	16.503(2)	13.7260(13)
b (Å)	22.583(4)	18.0513(19)	17.4178(15)
c (Å)	23.093(5)	17.1610(19)	21.3944(18)
α (°)	73.985(4)	90	90
β (°)	86.426(4)	105.059(3)	95.112(3)
γ (°)	83.187(4)	90	90
Volume ($Å^3$)/*Z*	5157.2(16) / 4	4936.7(10) / 4	5094.6(8) /4
ρ (Mg/m^3)	1.776	1.750	1.772
μ (Mo-Kα)/mm^{-1}	5.721	5.749	5.557
F(000)	2666.5	2523.2	2638
Crystal size (mm^3)	0.38x0.12x0.05	0.29x0.19x0.11	0.12x0.05x0.01
θ range (°)	2.10 to 28.40	2.26 to 28.35	2.180 to 28.298
Reflections collected	331123	160868	81564
Independent reflections [*R*(int)]	25804 [0.0395]	12314 [0.0563]	12662 [0.1969]
Final R_1, wR_2 [$I > 2\sigma(I)$]	0.0205, 0.0396	0.0312, 0.1059	0.0633, 0.1256
R_1, wR_2 (all data)[a]	0.0327, 0.0852	0.0417, 0.1190	0.1349, 0.1565
GOF (F^2)[b]	1.040	0.924	1.015
Largest diff. peak and hole/e.$Å^{-3}$	2.23 and -2.21	1.20 and -0.76	1.87 and -2.17

[a] $R_1 = \sum(|F_o| - |F_c|) / \sum |F_o|$. $wR_2 = [\sum w (F_o^2 - F_c^2)^2 / \sum w(F_o^2)^2]^{1/2}$. [b] Goodness-of-fit $= [\sum w (F_o^2 - F_c^2)^2 / (n_{obs} - n_{param})]^{1/2}$

2. Results and discussion

2.1 Spectra for characterization

a)

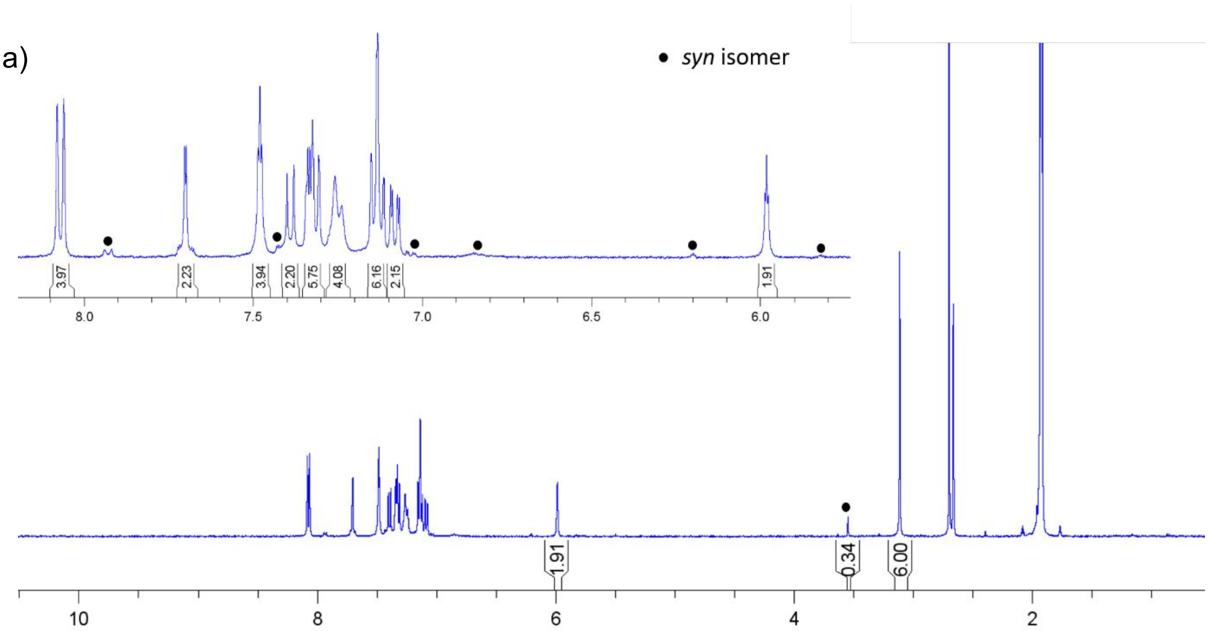

b)

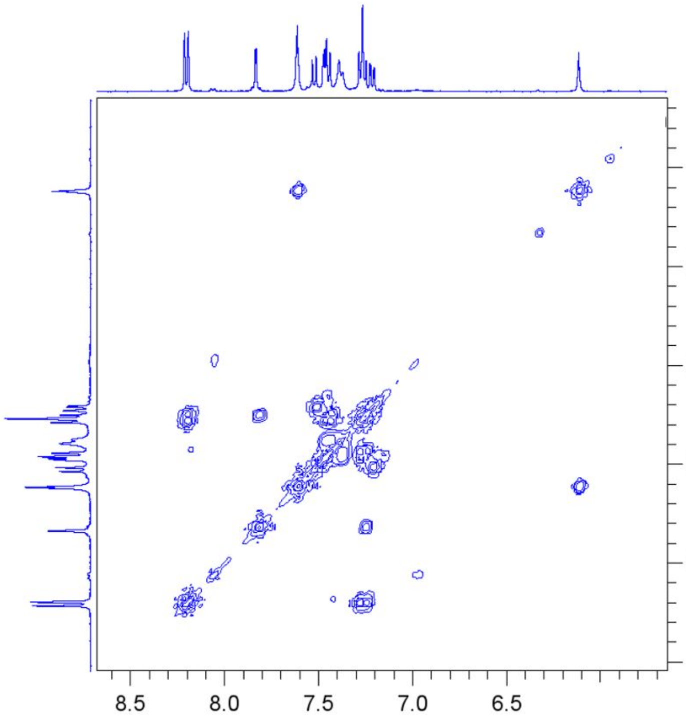

c)

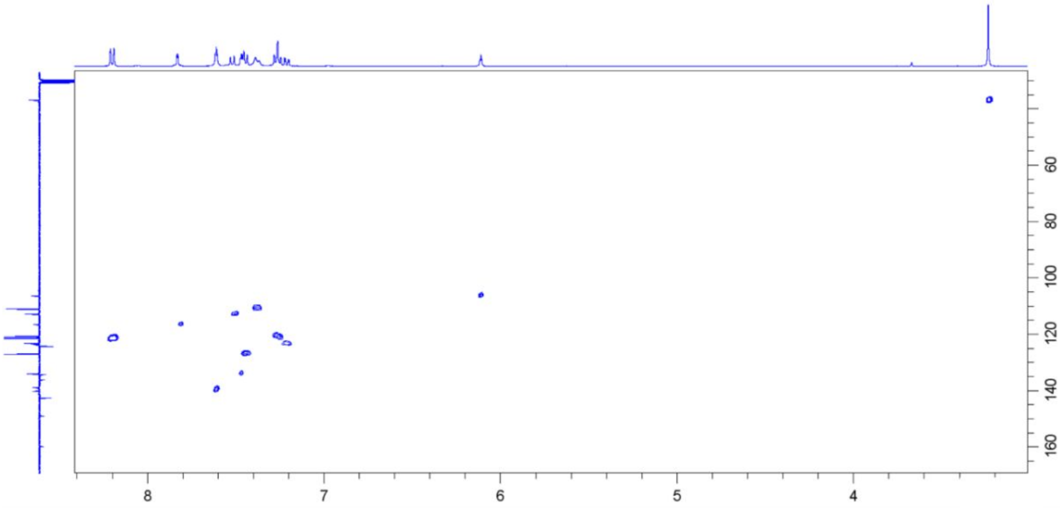

d)

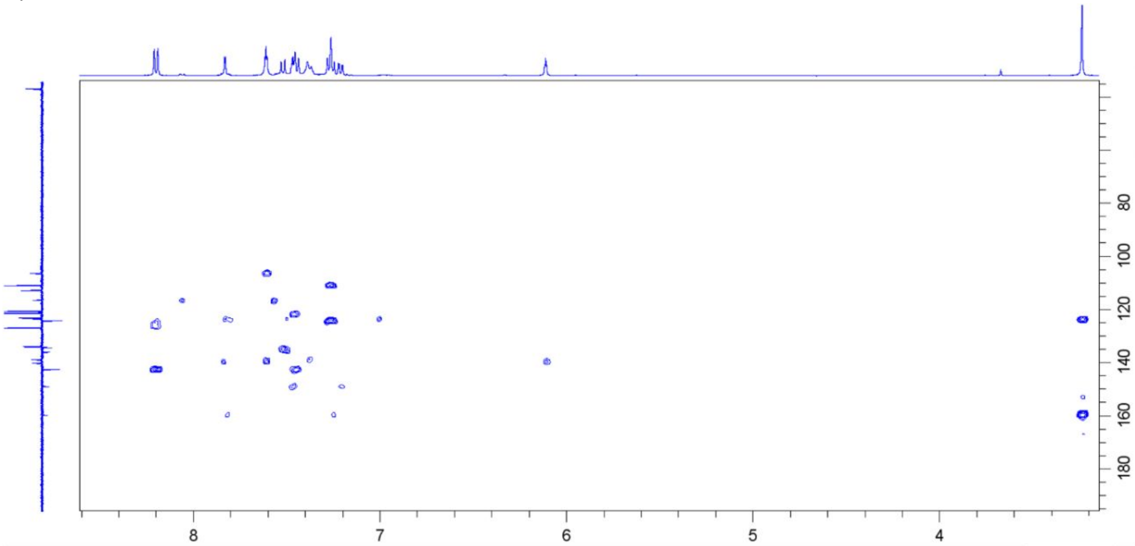

e)

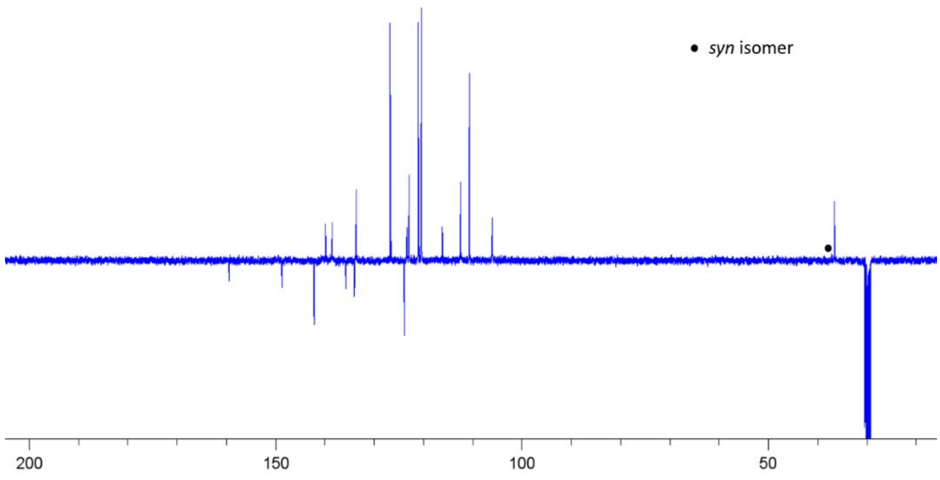

• syn isomer
200
150
100
50

f)

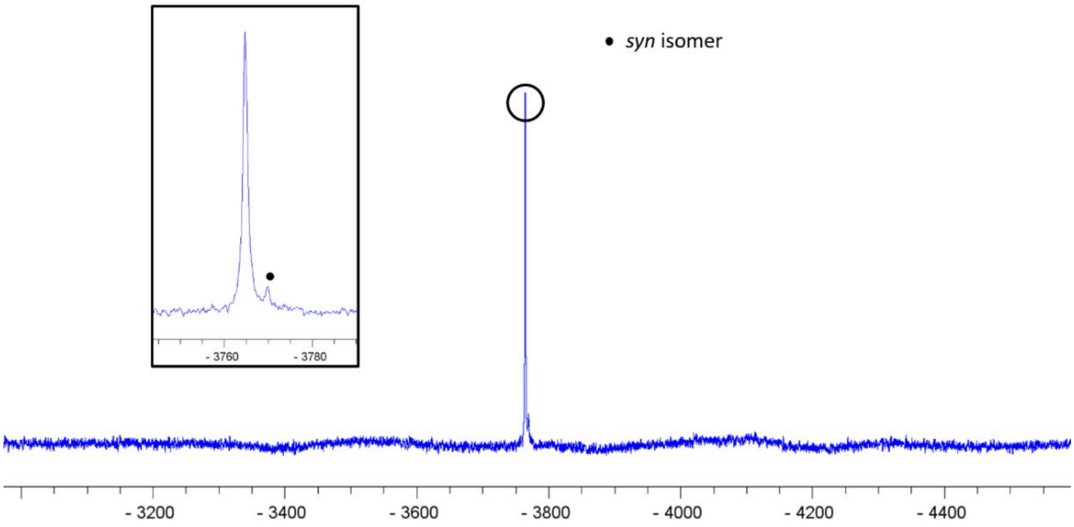

• syn isomer
- 3760
- 3780
- 3200
- 3400
- 3600
- 3800
- 4000
- 4200
- 4400

g)

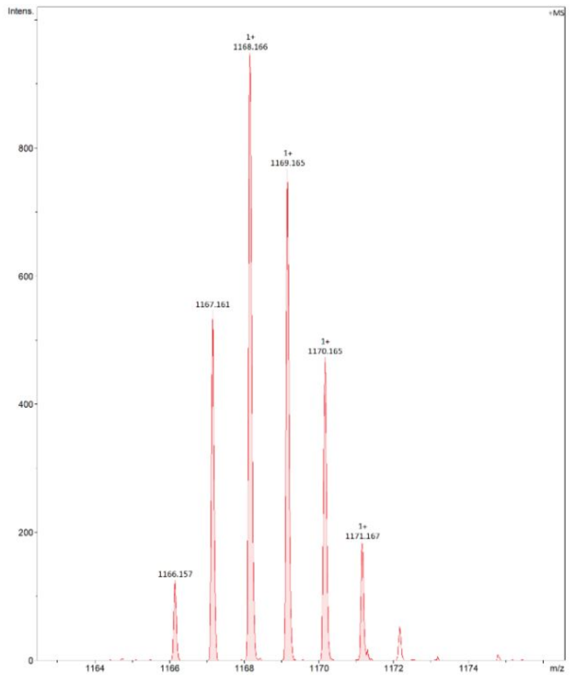

Figure S1. a) ^{1}H NMR spectrum of **1** in acetone-d_6; **b)** ^{1}H-^{1}H COSY NMR spectrum of **1** in acetone-d_6; **c)** ^{1}H-^{13}C HSQC NMR spectrum of **1** in acetone-d_6; **d)** ^{1}H-^{13}C HMBC NMR spectrum of **1** in acetone-d_6; **e)** ^{13}C{^{1}H} APT NMR spectrum of **1** in acetone-d_6; **f)** ^{195}Pt{^{1}H} NMR spectrum of **1** in acetone-d_6; **g)** Mass spectrum of **1**.

a)

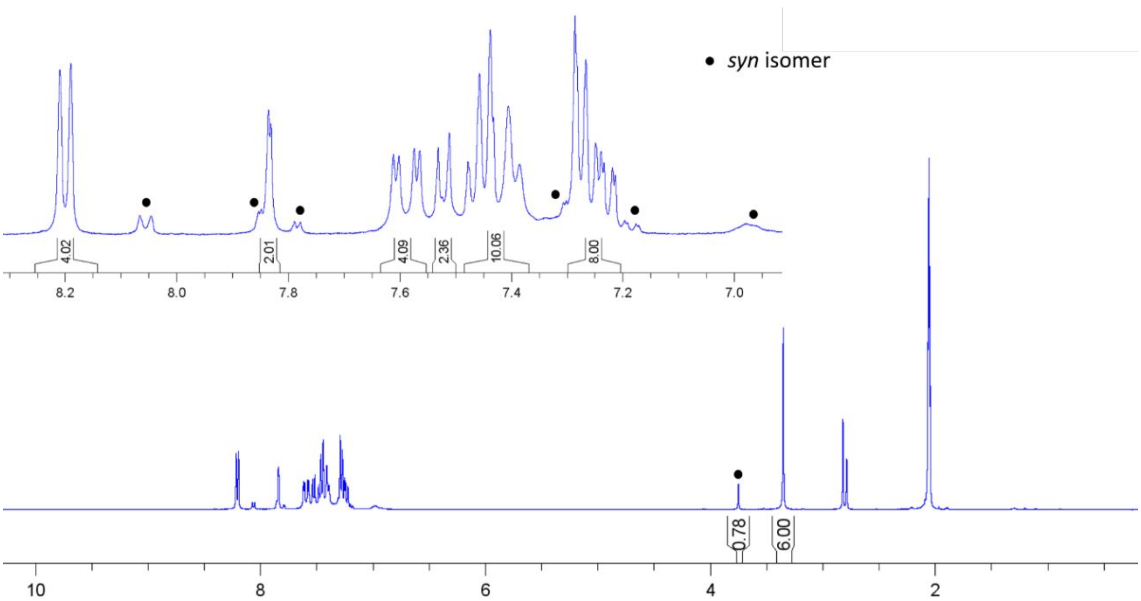

b)

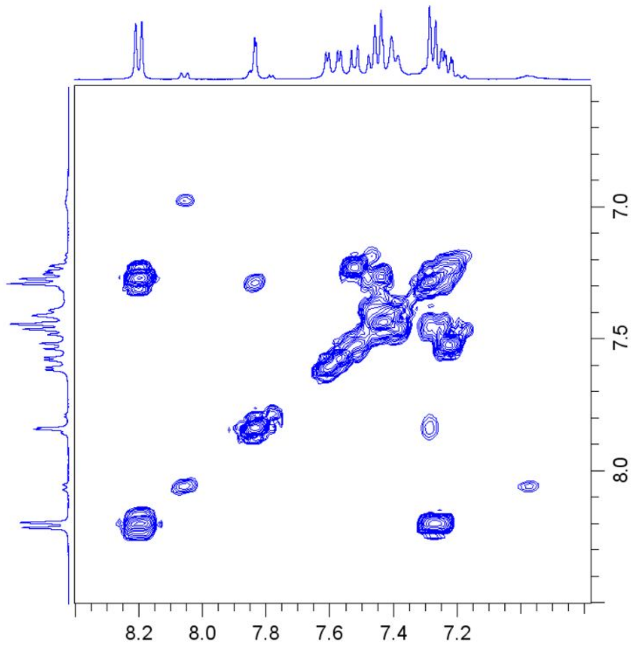

c)

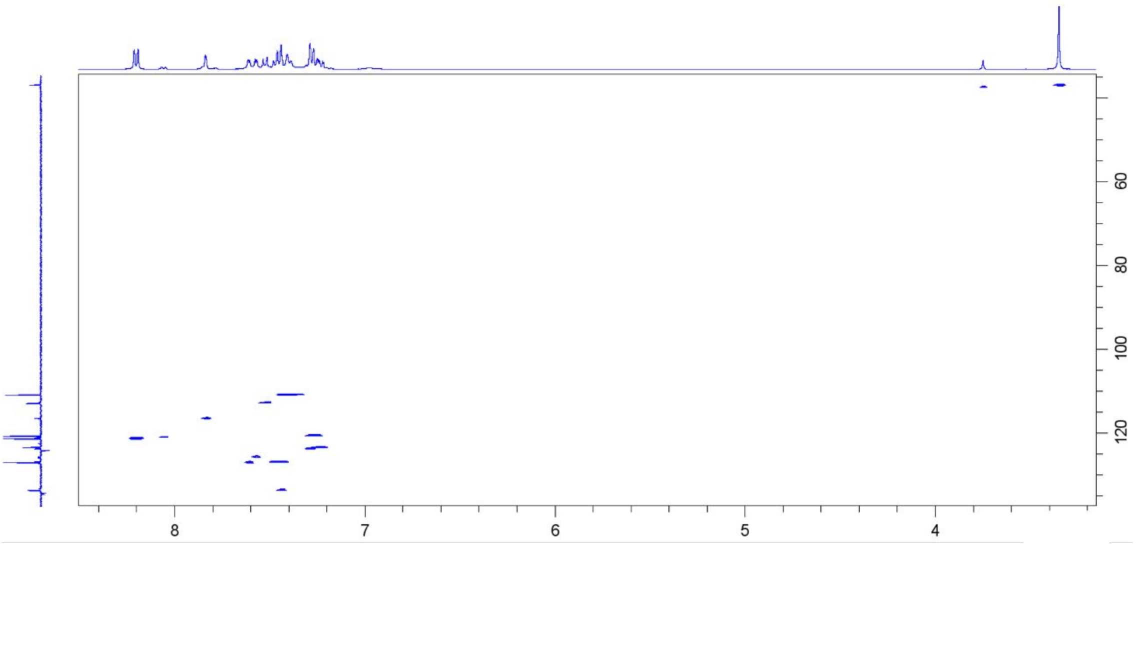

d)

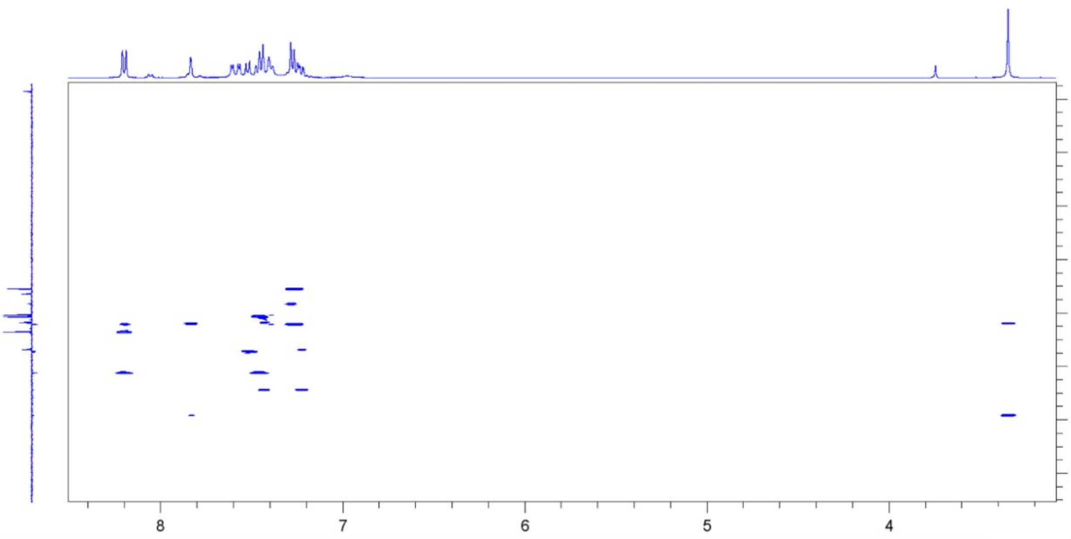

e)

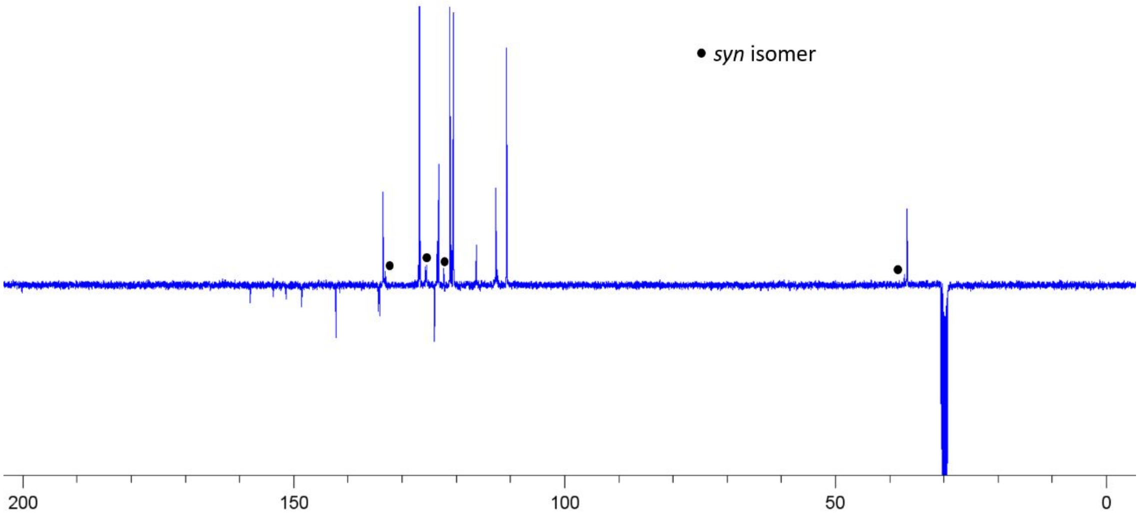

f)

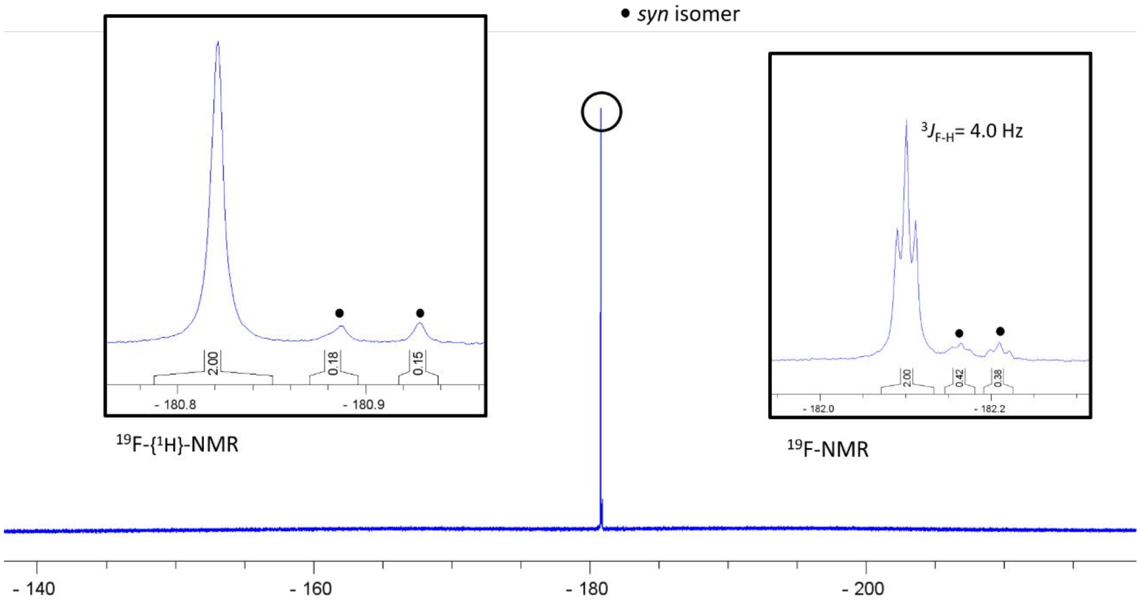
• syn isomer
2.00
0.18
0.15
- 180.8
- 180.9
19F-{1H}-NMR
3JF-H= 4.0 Hz
2.00
0.42
0.38
- 182.0
- 182.2
19F-NMR
- 140
- 160
- 180
- 200

g)

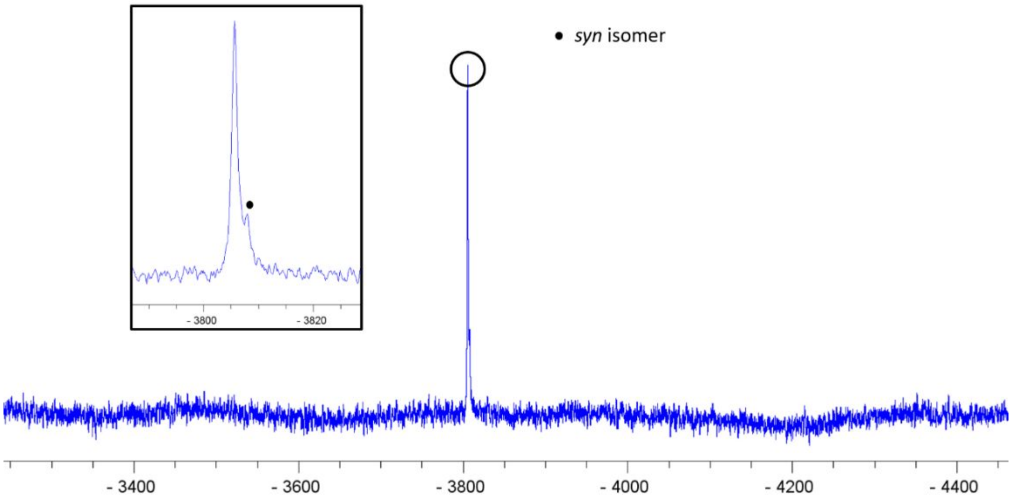
• syn isomer
- 3800
- 3820
- 3400
- 3600
- 3800
- 4000
- 4200
- 4400

h)

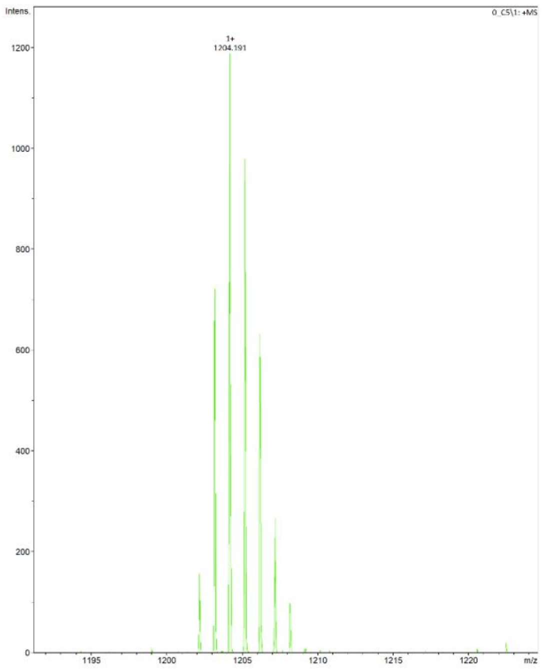

Figure S2. a) ^{1}H NMR spectrum of **2** in acetone-d_6; **b).** ^{1}H-^{1}H COSY NMR spectrum of **2** in acetone-d_6; **c)** ^{1}H-^{13}C HSQC NMR spectrum of **2** in acetone-d_6. **d)** ^{1}H-^{13}C HMBC NMR spectrum of **2** in acetone-d_6; **e)** $^{13}C\{^{1}H\}$ APT NMR spectrum of **2** in acetone-d_6; **f)** $^{19}F\{^{1}H\}$ spectrum of **2** in acetone-d_6 and expanded images of $^{19}F\{^{1}H\}$ (left) and ^{19}F NMR (right). **g)** $^{195}Pt\{^{1}H\}$ NMR spectrum of **2** in acetone-d_6; **h)** Mass spectrum of **2**.

a)

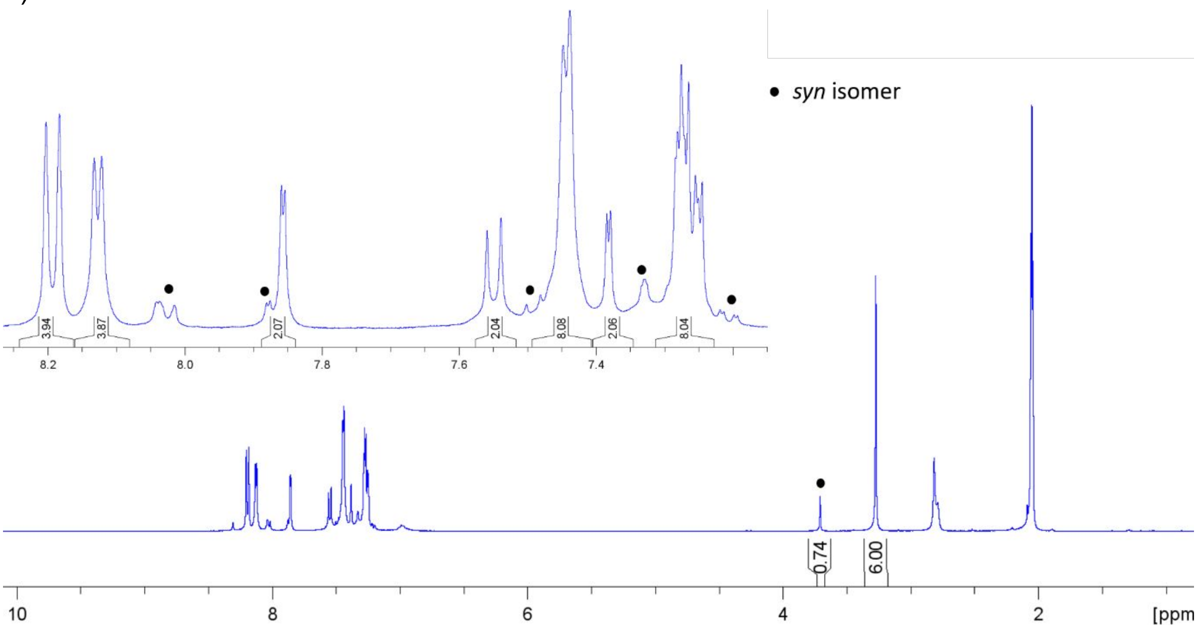

b)

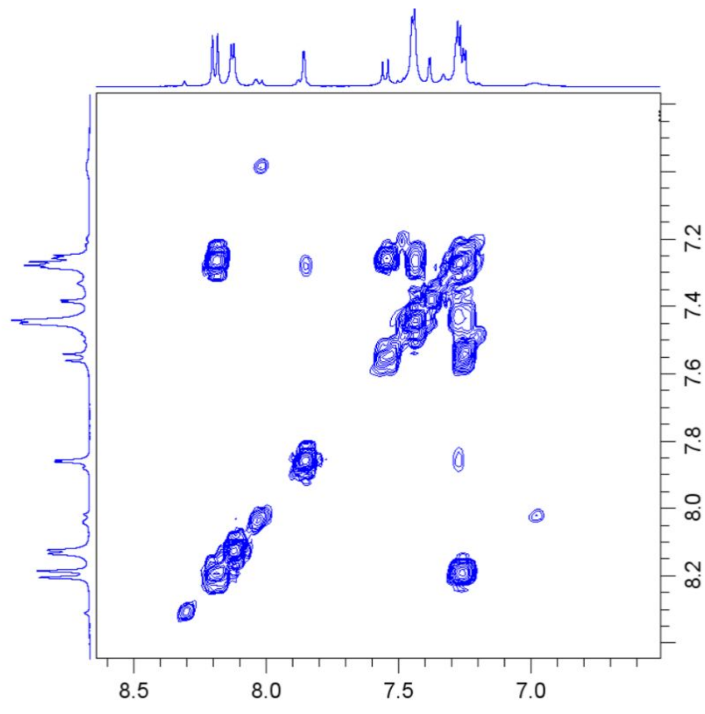

c)

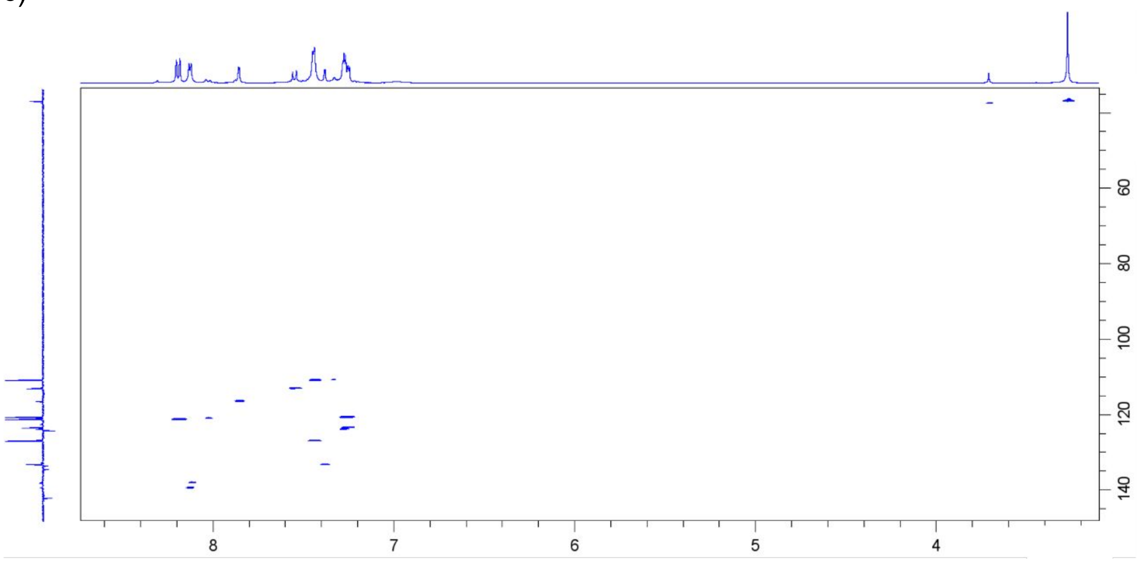

d)

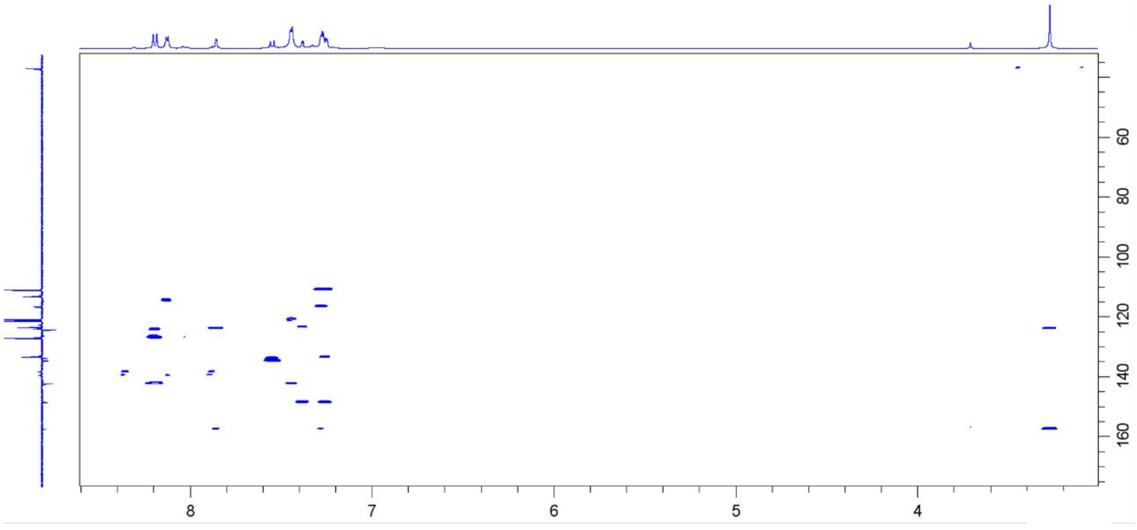

e)

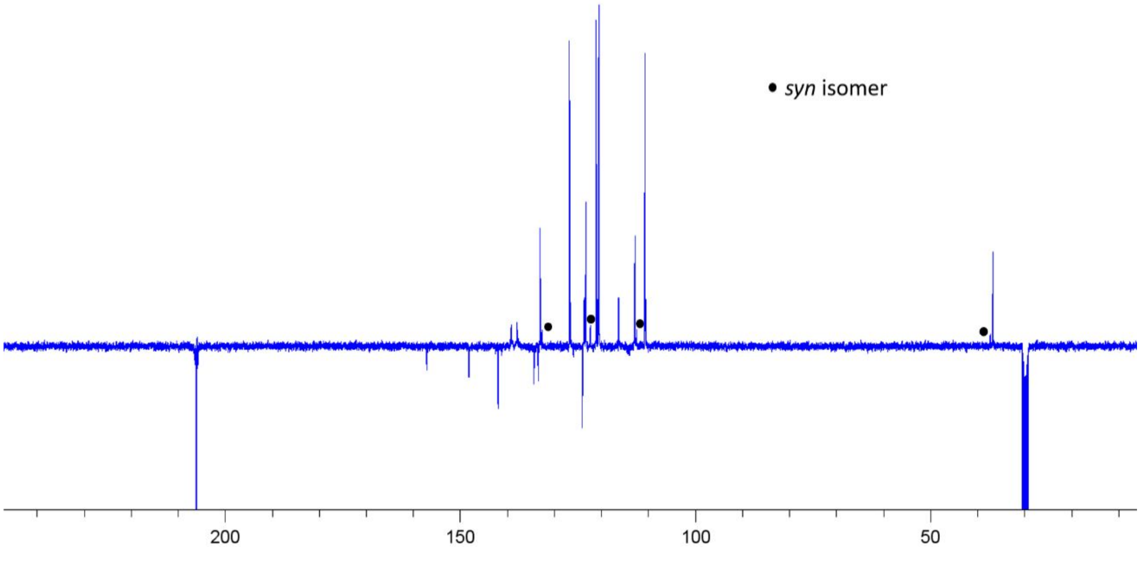

f)

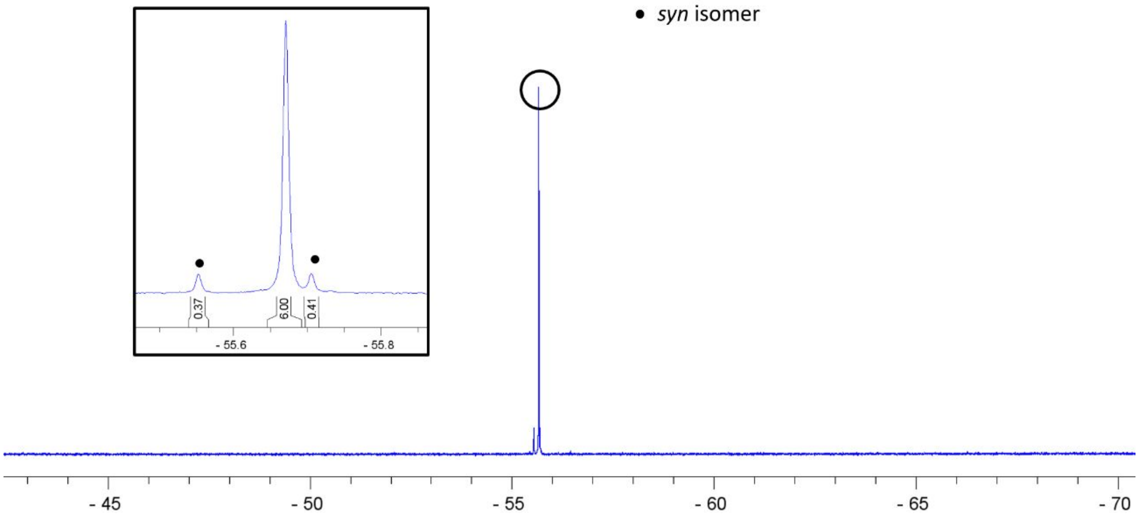

• syn isomer
0.37
6.00
0.41
- 55.6
- 55.8
- 45
- 50
- 55
- 60
- 65
- 70

g)

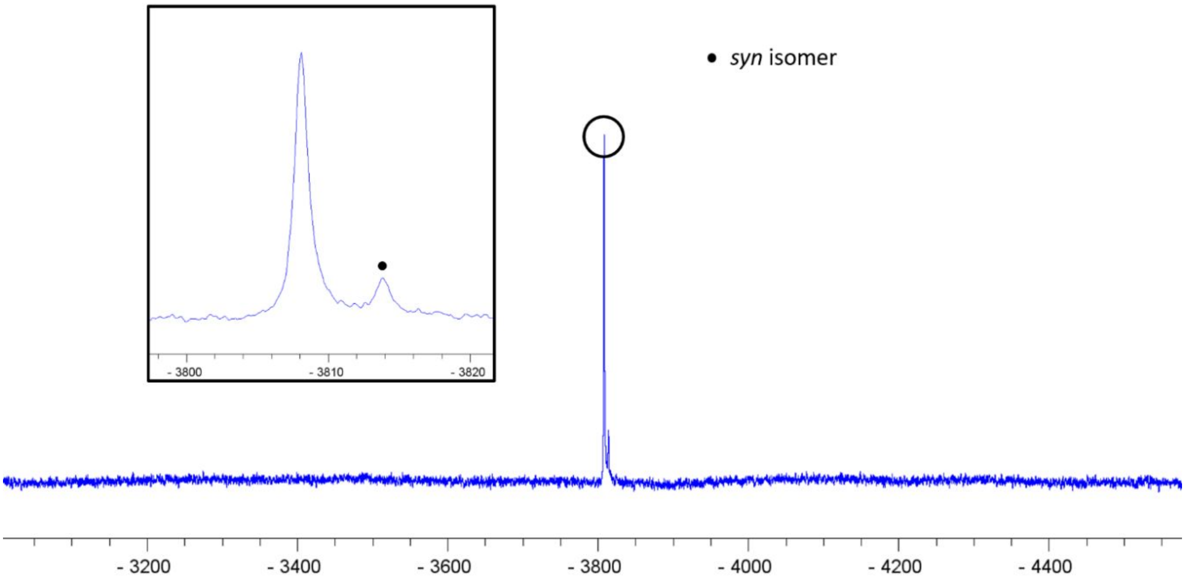

• syn isomer
- 3800
- 3810
- 3820
- 3200
- 3400
- 3600
- 3800
- 4000
- 4200
- 4400

h)

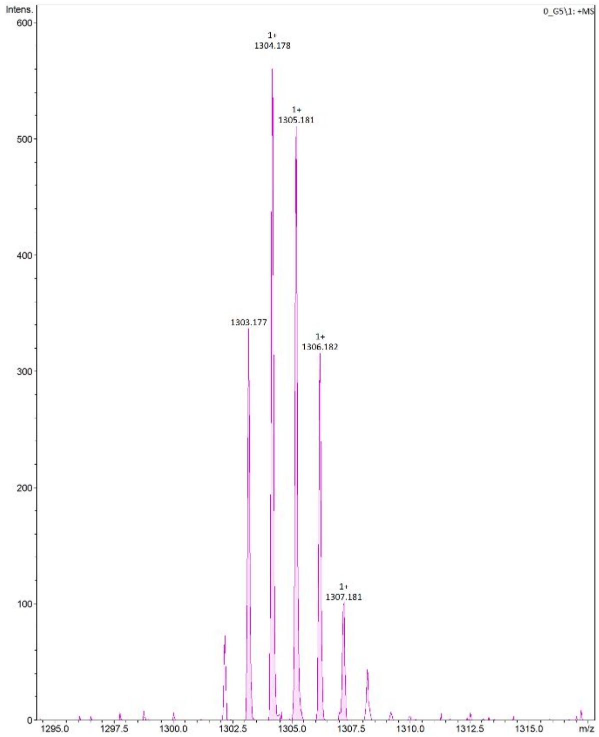

Figure S3. a) ^{1}H NMR spectrum of **3** in acetone-d_6; **b)** ^{1}H-^{1}H COSY NMR spectrum of **3** in acetone-d_6; **c)** ^{1}H-^{13}C HSQC NMR spectrum of **3** in acetone-d_6; **d)** ^{1}H-^{13}C HMBC NMR spectrum of **3** in acetone-d_6; **e).** ^{13}C{^{1}H} APT NMR spectrum of **3** in acetone-d_6; **f)** ^{19}F{^{1}H} spectrum of **3** in acetone-d_6; **g)** ^{195}Pt{^{1}H} NMR spectrum of **3** in acetone-d_6; **h)** Mass spectrum of **3**.

2.2 Full description of the X-ray molecular structures.

Complexes **1-*a*, 2-*a*, 3-*a*** are dinuclear species with a butterfly-like structure (see Figure S4). In case of compound **1-*a*** there are two molecules (**A**, **B**) in the asymmetric unit. In all cases, the molecules consist of two "Pt(Cz-C^C*$_{im}$)" metalocycles bridged by two Rpz ligands. They exhibit an *anti*-arrangement of the Pt-C* bonds, with C*-Pt-Pt'C*' torsion angles of 94.96 (2)^{o} **1-*a* A**, 83.50 (2) **1-*a* B**, 79.83 **2-*a*** and 84.15^{o} **3-*a*.**

The six-membered ring Pt_2N_4 has the typical boat-like conformation with an angle between the Pt-N-N-Pt fragments of about 80° [80.59^{o} (**1-*a* A**), 79.70^{o} (**1-*a* B**, 80.62^{o} (**2-*a***), 82.82^{o} (**3-*a***)] and an angle between the best least-squares planes of the platinum environments of 78.81^{o} (**1-*a* A**), 78.09^{o} (**1-*a* B**), 77.05^{o} (**2-*a***) and 71.35^{o} (**3-*a***). In the

dinuclear molecules, each Pt center lies in a distorted square planar coordination environment as a consequence of the small bite angle of the Cz-C^C* cyclometalated ligand of about 80º (Table S2).

Table S2: Selected bond lengths (Å) and angles (º) for **1-*a*, 2-*a*, 3-*a***

Bond lengths (Å)	**1-*a* (A)**	**1-*a* (B)**	**2-*a***	**3-*a***
Pt1-C1	1.967(4)	1.974(4)	1.938(5)	1.957(11)
Pt1-C5	2.005(4)	2.020(4)	2.038(5)	1.990(9)
Pt1-N7	2.091(3)	2.071(4)	2.082(4)	2.101(8)
Pt1-N9	2.057(3)	2.051(3)	2.058(4)	2.058(8)
Pt2-C23	1.967(4)	1.952(4)	1.977(4)	1.956(11)
Pt2-C27	1.998(4)	2.013(4)	1.995(4)	2.008(10)
Pt2-N8	2.054(4)	2.056(3)	2.027(4)	2.070(9)
Pt2-N10	2.078(3)	2.085(3)	2.095(3)	2.098(8)
Pt···Pt	3.2998(4)	3.2821(6)	3.2599(3)	3.1762
Bond angles(º)	**1-*a* (A)**	**1-*a* (B)**	**2-*a***	**3-*a***
C1-Pt1-C5	80.31(16)	78.97(18)	79.6(2)	80.5(4)
C1-Pt1-N7	99.72(14)	101.10(17)	103.3(2)	100.3(4)
C5-Pt1-N9	95.19(14)	94.56(15)	91.95(19)	94.1(3)
N7-Pt1-N9	84.80(13)	85.53(14)	85.18(14)	85.1(3)
C23-Pt2-C27	79.92(17)	80.27(16)	79.90(18)	79.9(4)
C23-Pt2-N10	99.67(16)	98.84(15)	100.66(16)	101.0(4)
C27-Pt2-N8	95.84(15)	95.74(15)	94.39(16)	94.5(4)
N8-Pt2-N10	84.57(14)	84.74(13)	85.14(14)	84.7(3)
C9-N3-C11	125.3(3)	124.0(4)	127.2(4)	123.0(9)
C9-N3-C22	126.0(3)	127.5(4)	124.6(6)	126.2(9)
C11-N3-C22	108.8(3)	108.4(4)	108.1(6)	109.4(9)
C31-N6-C33	126.8(4)	125.1(4)	124.5(4)	125.0(8)
C31-N6-C44	124.1(4)	126.7(4)	126.4(4)	126.5(9)
C33-N6-C44	108.9(4)	108.2(3)	108.5(4)	108.0(9)

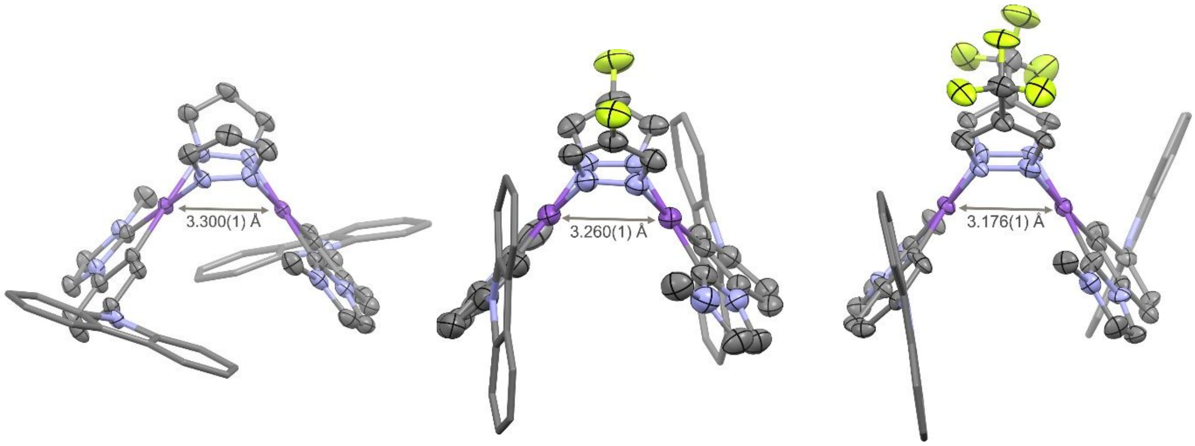

(a) (b) (c)

Figure S4. Molecular structures of **1-*a* A** (a), **2-*a*** (b), **3-*a*** (c). Solvent molecules and hydrogen atoms have been omitted for clarity.

These angles together with the Pt–C_{Ar} and Pt–C* distances are similar to those found in other compounds containing five-membered cycloplatinated N-heterocyclic carbenes.[1,2] The Pt-N bond distances are also similar to those found in dinuclear bis-pyrazolate-bridge complexes containing ligands with high *trans* influence.[2,3]

In these complexes, the intermetallic separation decreases with the presence of electro-withdrawing substituents on the pyrazolate bridging groups [3.2998(4) Å (**1-*a* A**), 3.2821(6)) Å (**1-*a* B**) > 3.2599(1) Å **(2-*a*)** > 3.1762 Å **(3-*a*)]** (see Table S2). In all cases, they are in the range of those found in analogous compounds with EtO_2C-C^C* as cyclometalated NHC carbene,[2-4] big enough to ensure the absence of a metal-metal bond,[5, 6] but not to preclude some degree of intermetallic interaction.[7, 8]

The packing arrangement of these complexes show the existence of not significant π-π intermolecular interactions (Figure S5).

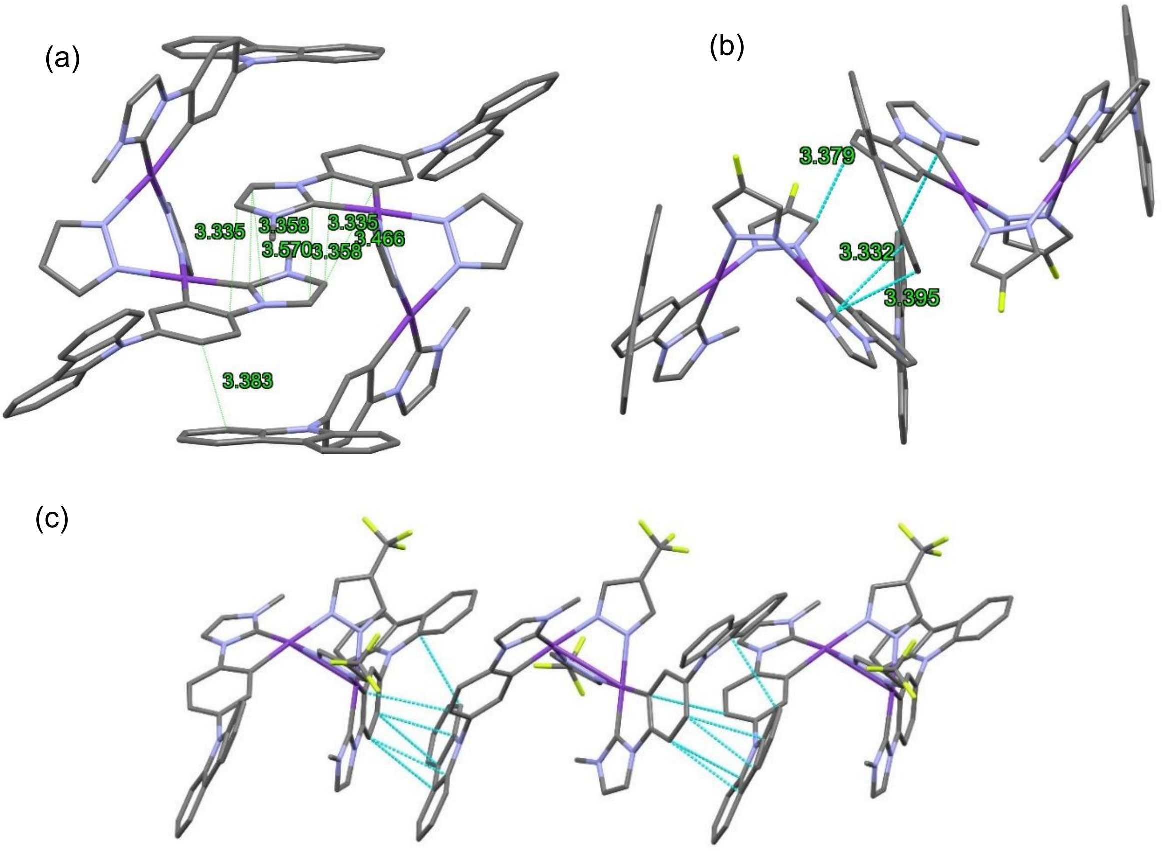

Figure S5. Supramolecular structures of **1-*a*** (a), **2-*a*** (b), **3-*a*** (c).

2.3 TGA analysis

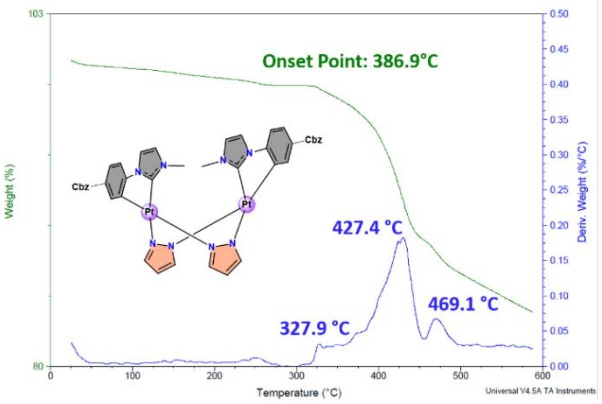

(a)

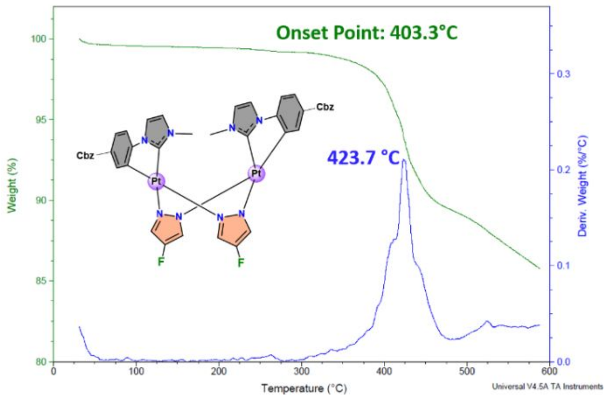

(b)

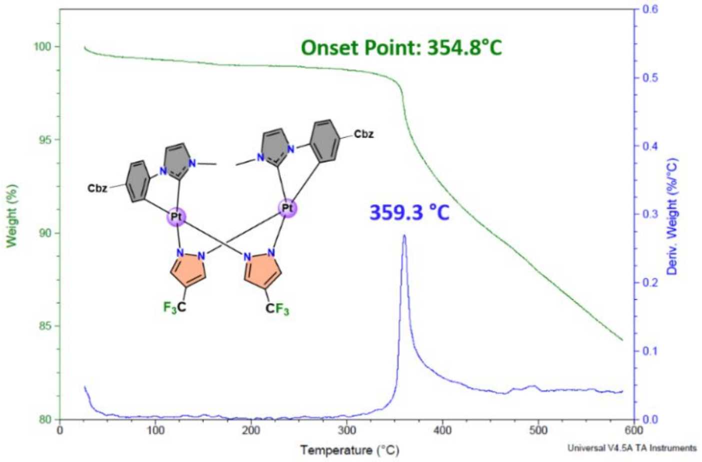

(c)

Figure S6.TGA (green trace, vertical scale on the left) and DTA (blue trace, vertical scale on the right) of compound **1** (a), **2** (b) and **3** (c) recorded on heating at 10 °C min^{-1} under N_2 atmosphere.

2.4 Computational results

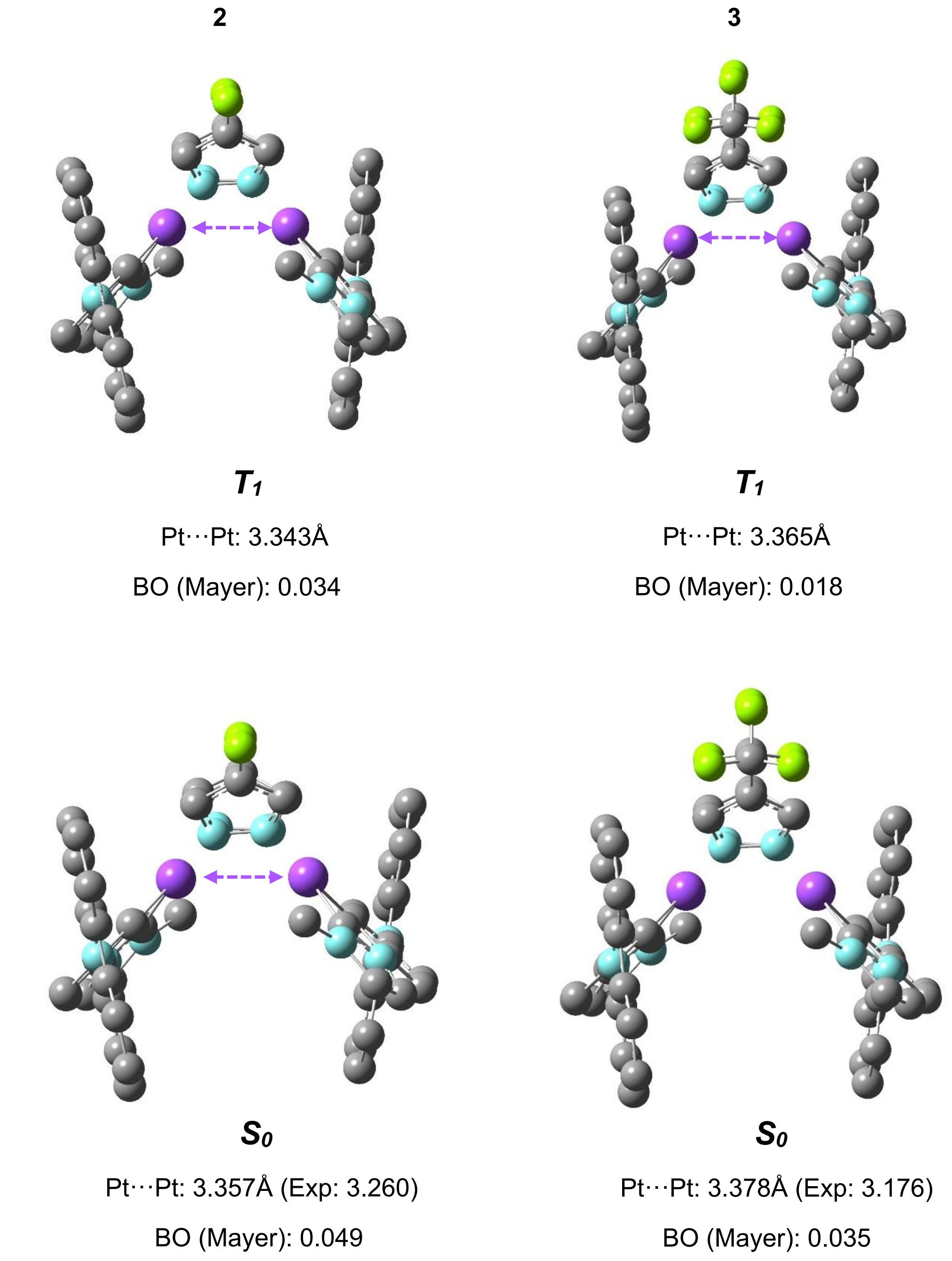

Figure S7: DFT (S_0) and UDFT (T_1) optimized structures for **2-a** and **3-a**.

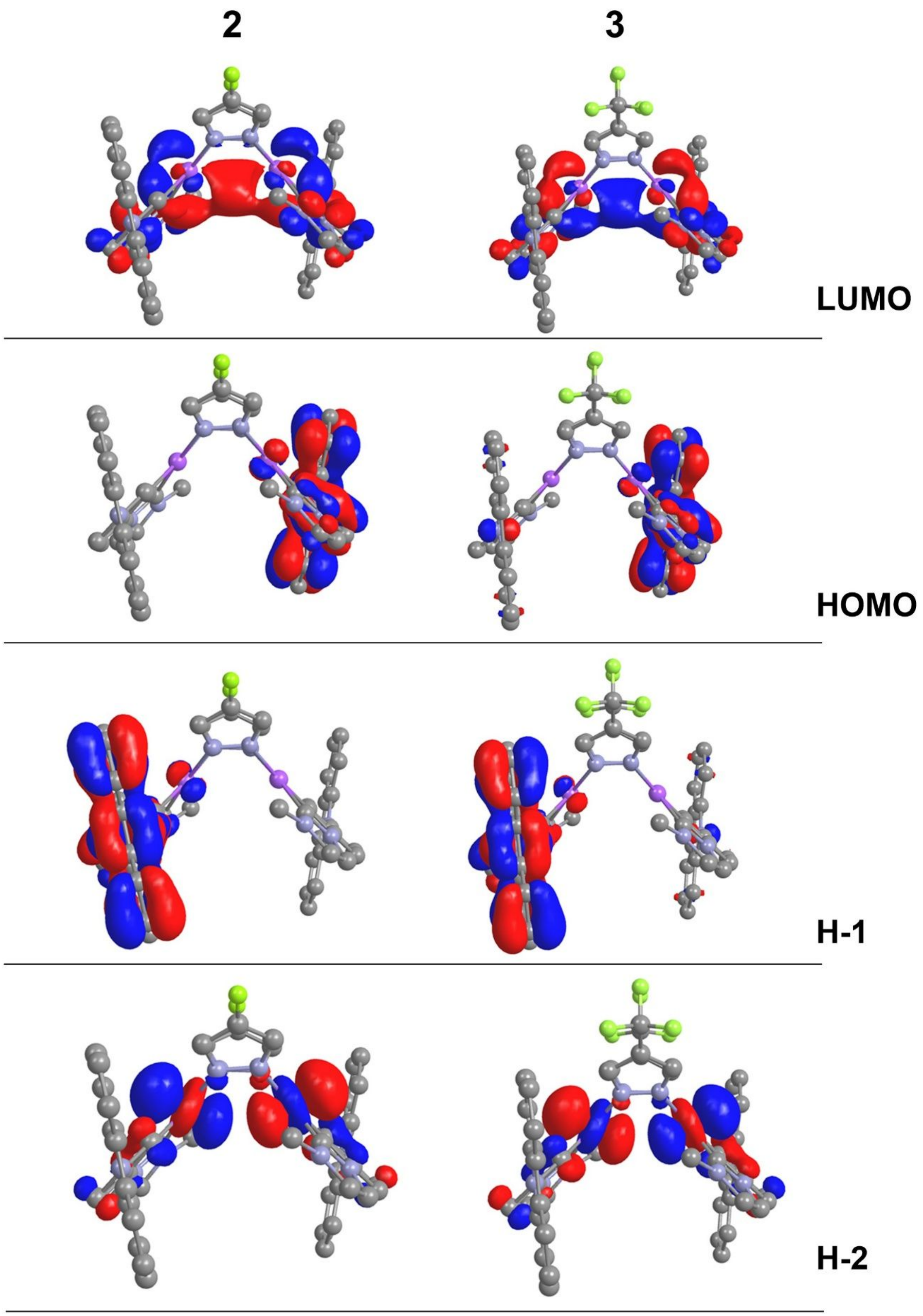

Figure S8. Frontier Molecular orbital plots (isovalue 0.03) for **2-a** and **3-a**.

Table S3. Population analysis (%) of MOs in the S_0 in THF solution.

MO	eV		Pt		NHC [Cz]		Rpz	
	**2-a**	**3-a**	**2-a**	**3-a**	**2-a**	**3-a**	**2-a**	**3-a**
**LUMO**	-1.28	-1.32	39	38	60[2]	60[2]	1	1
**HOMO**	-5.7	-5.7	3	2	97[73]	97[73]	1	0
**H_{-1}**	-5.7	-5.7	3	2	97[73]	97[73]	1	0
**H_{-2}**	-6.02	-6.12	77	75	19[3]	22[4]	4	3

Table S4. Vertical absorptions (S_n) and emission (T_1) energies calculated by DFT (TD-DFT) for **2-a** and **3-a** in THF from the optimized S_0.

Comp.		λ_{calc} (nm)	o.s.	Transition (%)	Assignment.
**2-a**	S_1	343.8	0.0491	$H_{-1}\rightarrow L$ (31%)<br>$H\rightarrow L$ (20%)<br>$H_{-2}\rightarrow L$ (22%)	ILCT/LMCT
	S_2	343.0	0.0001		
	S_3	333.9	0.2721	$H_{-2}\rightarrow L$(72%)	MLCT
	T_1	419.2	0	$H\rightarrow L$ (10%)<br>$H_{-1}\rightarrow L$ (16%)	
**3-a**	S_1	344.8	0.0001		
	S_2	344.5	0.0974	$H_{-1}\rightarrow L$ (50%)<br>$H\rightarrow L$ (15%)	ILCT/LMCT
	S_3	330.5	0.2687	$H_{-2}\rightarrow L$ (86%)	MLCT
	T_1	421.29	0	$H\rightarrow L_{+1}$ (12%)<br>$H_{-1}\rightarrow L$ (21%)<br>$H_{-6}\rightarrow L$ (10%)	

2.5 Photophysical and electrochemical properties

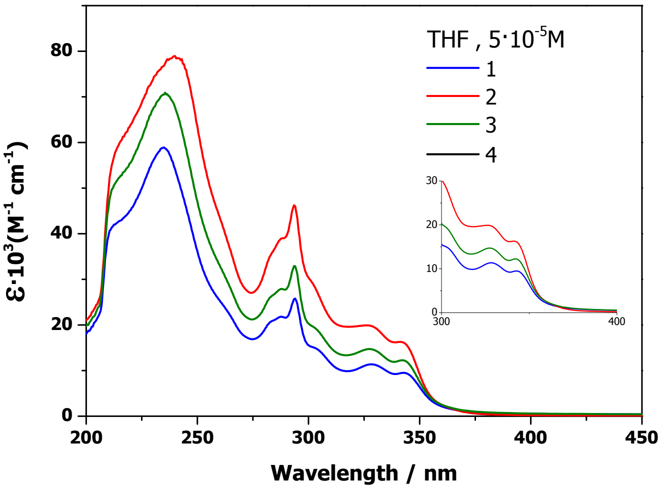

Figure S9. UV-Vis spectra of complexes **1-3** in THF solutions (5·10^{-5} M).

Table S5. Absorption data for **1-3** in THF solution at 298 K.

Comp.	λ_{abs} /nm (10^3 ε M^{-1} cm^{-1})
**1**	235(58.92), 284_{sh} (20.94), 294 (25.78), 304_{sh} (14.69), 328(11.37), 343 (9.51)
**2**	236(70.52), 284_{sh} (26.51), 294 (32.89), 304_{sh} (18.89), 327(14.71), 343 (12.21)
**3**	240(78.94), 285_{sh} (36.89), 293 (45.54), 304_{sh} (27.19), 327(19.89), 341 (16.28)

5·10^{-5} M (pathlength 1 cm)

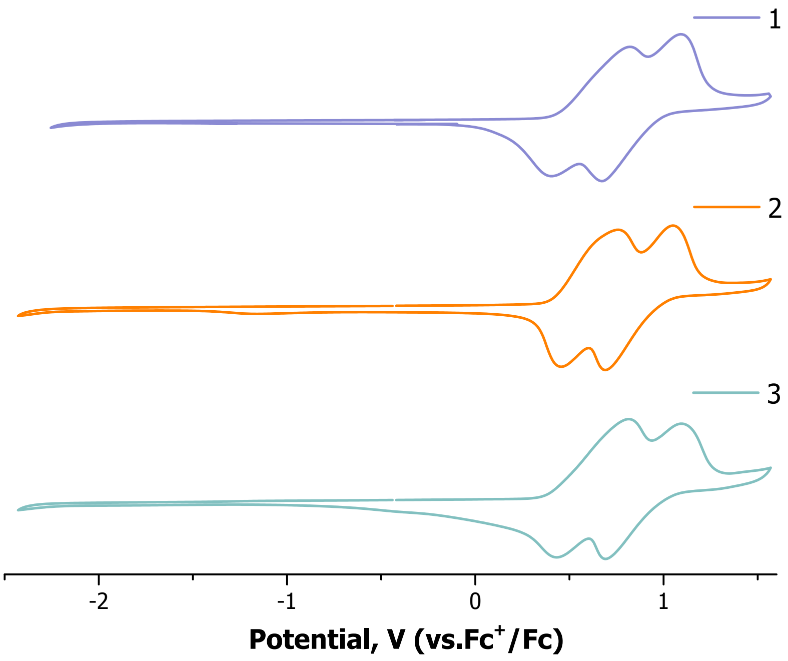

Figure S10. Cyclic voltammograms of complexes **1-3** in CH_2Cl_2 at 100 mV s^{-1}

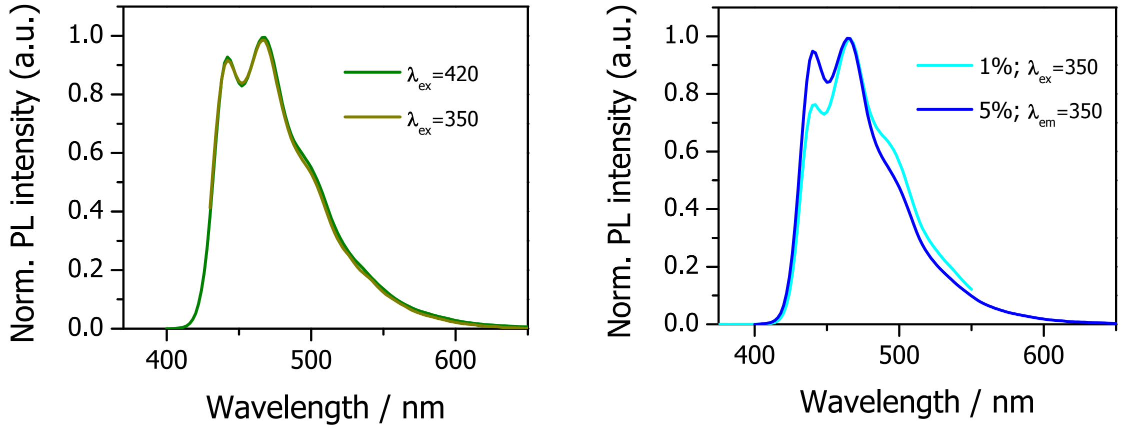

Figure S11. Emission spectra of complex **2** in PMMA films at 5wt.% upon different excitation wavelength (left) and complex **1** in PMMA films at different doping concentrations (right).

2.6 Electroluminescence

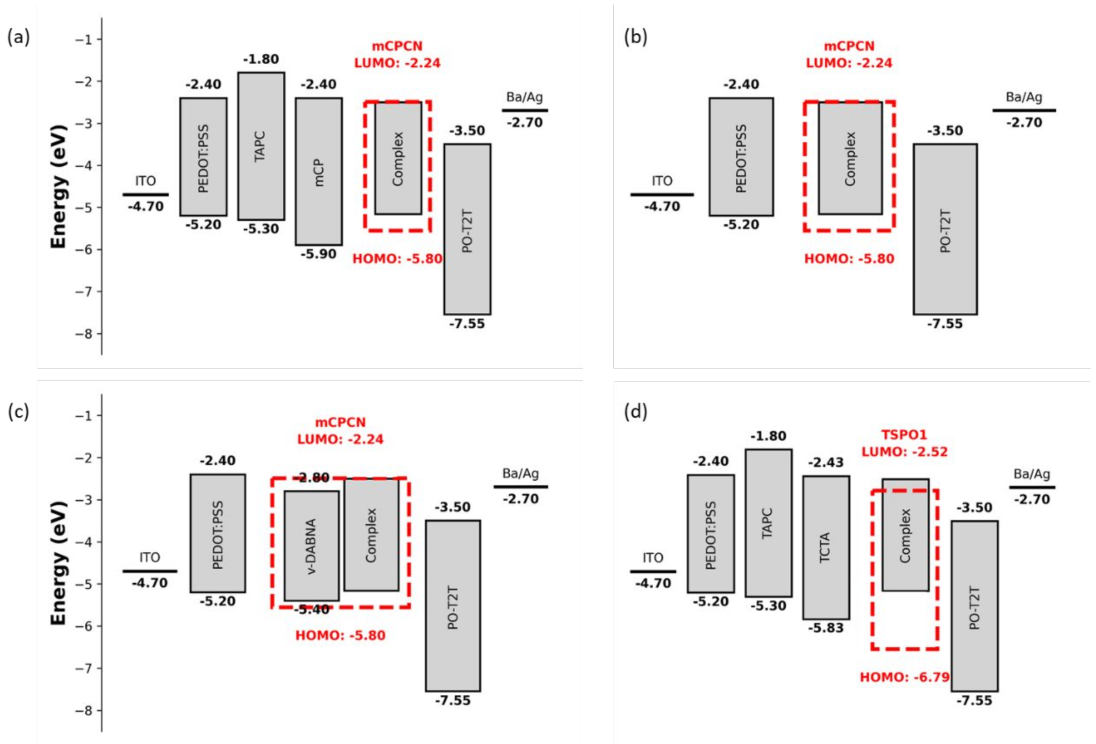

Figure S12. a-d) Energy level diagrams of the devices fabricated in this work.

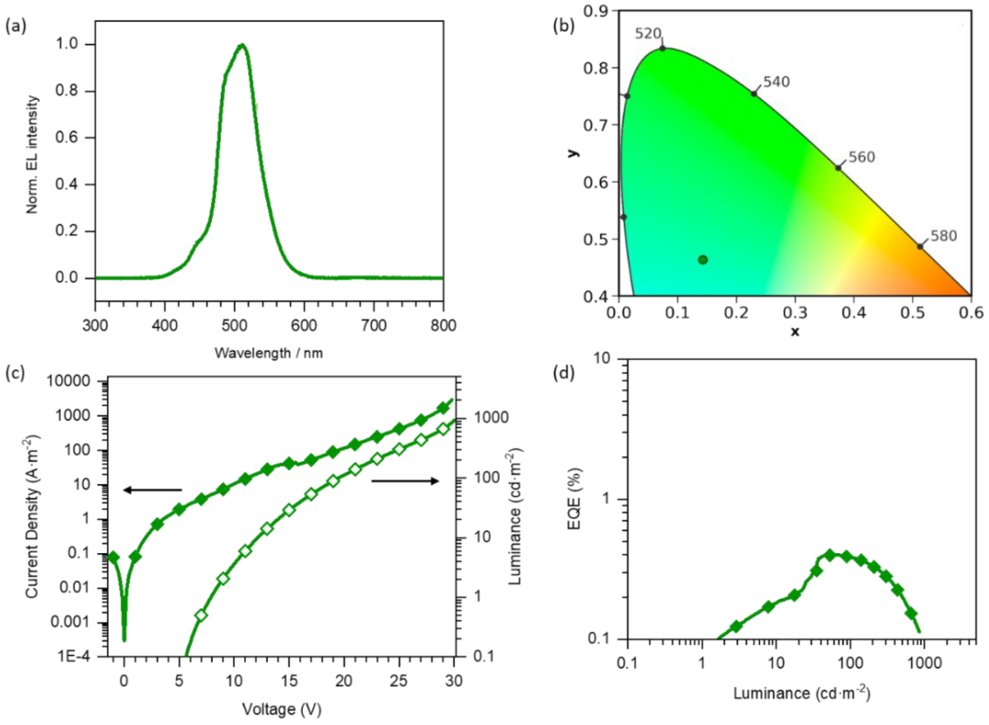

Figure S13. Thermally evaporated PhOLED with mCPCN and complex **1** as host and guest, respectively. (a) Electroluminescent spectra, (b) color points in the chromaticity diagram, (c) *J-V-L* characteristics and (d) EQE versus luminance plot.

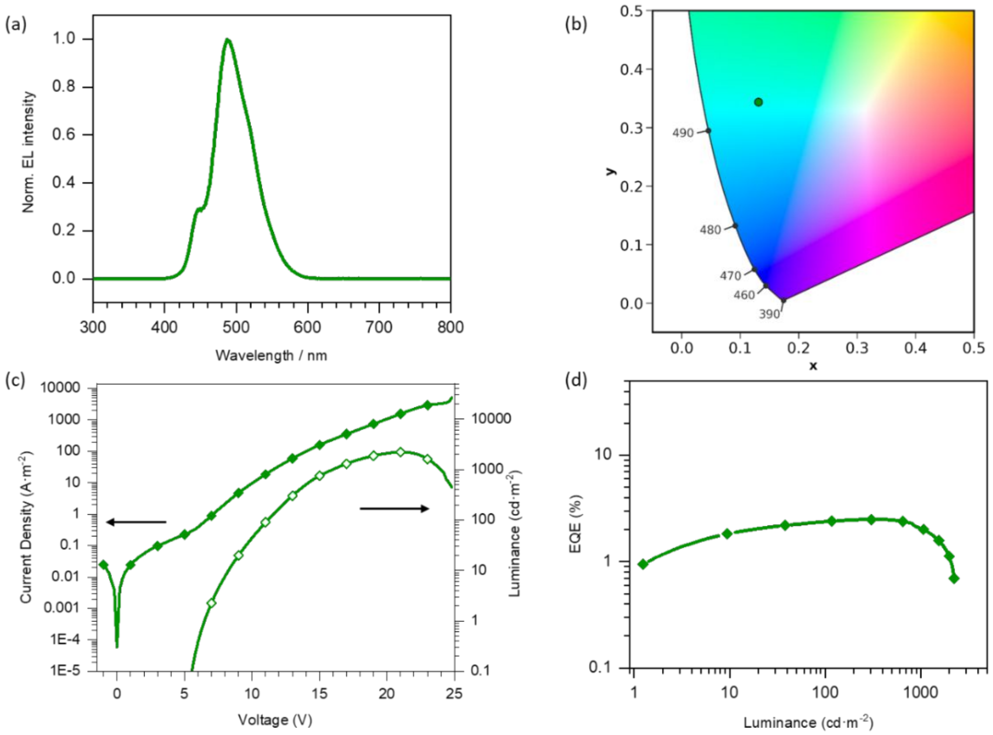

Figure S14. Solution processed PhOLED with mCPCN and complex **1** as host and guest, respectively. (a) Electroluminescent spectra, (b) color points in the chromaticity diagram, (c) *J-V-L* characteristics and (d) EQE versus luminance plot.

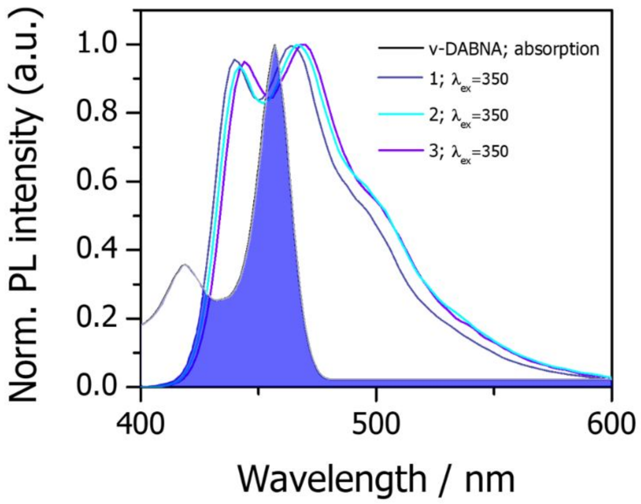

Figure S15. Absorption spectrum of solution 10^{-2} M of v-DABNA in toluene and PL spectra of complexes **1-3** in PMMA 5 wt%.

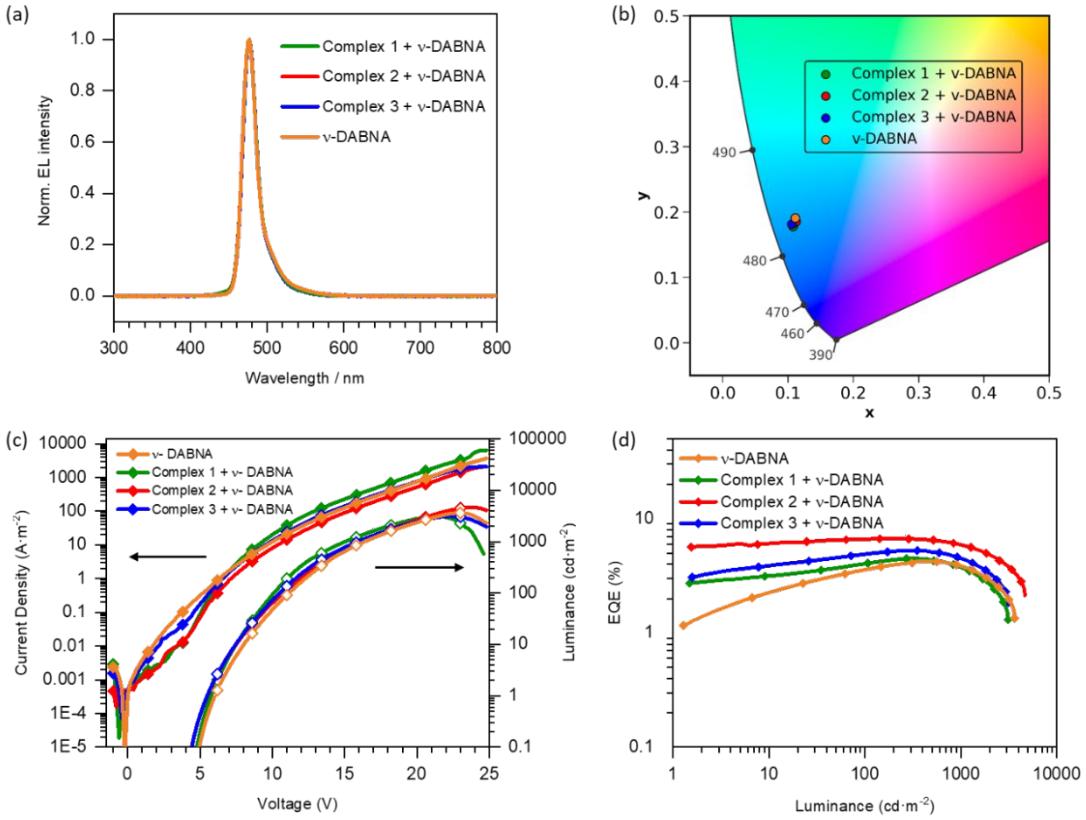

Figure S16. Solution processed OLEDs with tertiary system where mCPCN, complex **1-3** and v-DABNA are the host, sensitizer and terminal emitter, respectively. (a) Electroluminescent spectra, (b) color points in the chromaticity diagram, (c) *J-V-L* characteristics and (d) EQE versus luminance plot.

Table S6. Performance summary of the OLEDs described in this study. The EL data refers to devices operated at 8.0 to 10.0 V.

<table>
<tr><th>Host</th><th>Process</th><th>Emitter</th><th>λ_{EL} (nm)</th><th>CIE (x, y)</th><th>V_{on} (V)</th><th>L_{max} ($cd\cdot m^{-2}$)</th><th>CE ($cd\cdot A^{-1}$)</th><th>EQE (%)</th></tr>
<tr><td rowspan="7">mCPCN</td><td>Thermally evaporated</td><td>Complex 1</td><td>450_{sh}
490_{sh}
510_{max}</td><td>0.14, 0.46</td><td>8.9</td><td>949.2</td><td>1.01</td><td>0.40</td></tr>
<tr><td rowspan="6">Solution</td><td>Complex 1</td><td>450
489_{max}
510_{sh}</td><td>0.13, 0.34</td><td>6.5</td><td>2219.9</td><td>5.13</td><td>2.50</td></tr>
<tr><td>ν-DABNA</td><td>476_{max}</td><td>0.11, 0.19</td><td>6.0</td><td>3637.0</td><td>5.18</td><td>4.25</td></tr>
<tr><td>Complex 1 + ν-DABNA</td><td>476_{max}</td><td>0.11, 0.18</td><td>5.7</td><td>3107.7</td><td>5.16</td><td>4.48</td></tr>
<tr><td>Complex 2 + ν -DABNA</td><td>476_{max}</td><td>0.11, 0.19</td><td>5.5</td><td>4646.1</td><td>8.08</td><td>6.71</td></tr>
<tr><td>Complex 3 + ν -DABNA</td><td>476_{max}</td><td>0.11, 0.18</td><td>5.5</td><td>3133.5</td><td>5.95</td><td>5.27</td></tr>
<tr><td rowspan="3">TSPO1</td><td rowspan="3">Thermally evaporated</td><td>Complex 1</td><td>446
485_{sh}
503_{max}</td><td>0.14, 0.46</td><td>9.5</td><td>2357.7</td><td>10.72</td><td>4.26</td></tr>
<tr><td>Complex 2</td><td>446
509_{max}</td><td>0.15, 0.37</td><td>9.5</td><td>1093.9</td><td>7.91</td><td>3.60</td></tr>
<tr><td>Complex 3</td><td>446
509_{max}</td><td>0.17, 0.44</td><td>14.2</td><td>1261.8</td><td>10.27</td><td>4.02</td></tr>
</table>

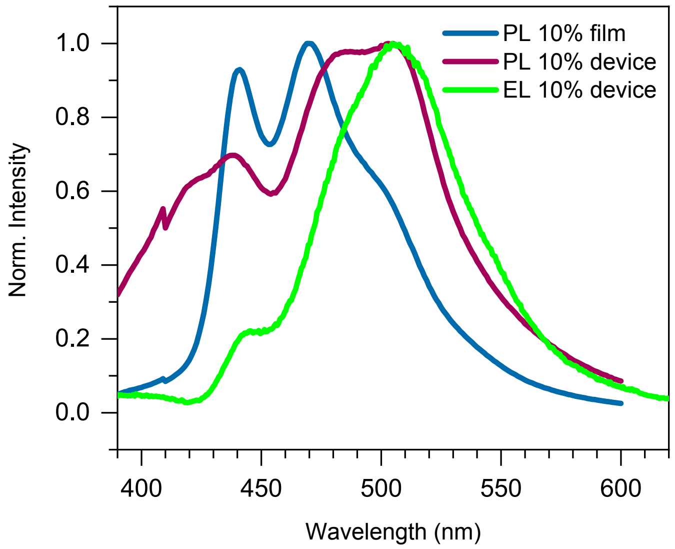

Figure S17. Photoluminescence of OLEDs with an emissive layer of TSPO1:**1** (10%). For convenience, the EL spectrum of the same devices and the PL spectrum of the film on glass have also been added.

3. REFERENCES

1. Arnal, L.; Fuertes, S.; Martin, A.; Sicilia, V., The Use of Cyclometalated NHCs and Pyrazoles for the Developement of Fully Efficient Blue Pt(II) Emitters and Pt/Ag Clusters. *Chem. Eur. J.* **2018,** *24*, 9377-9384.

2. Arnal, L.; Fuertes, S.; Martín, A.; Baya, M.; Sicilia, V., A Cyclometalated N-Heterocyclic Carbene: The Wings of the First Pt2(II,II) Butterfly Oxidized by CHI_3. *Chem. Eur. J.* **2018,** *24*, 18743-18748 and references therein.

3. Sicilia, V.; Arnal, L.; Escudero, D.; Fuertes, S.; Martín, A., Chamaleonic Photo- and Mechanoluminescence in Pyrazolate-Bridged NHC Cyclometalated Platinum Complexes. *Inorg. Chem.* **2021,** *60*, 12274-12284.

4. Pinter, P.; Unger, Y.; Strassner, T., Cyclometalated NHC Platinum(II) Complexes with Bridging Pyrazolates: Enhanced Photophysics of Binuclear Blue Emitters. *Chem. Photo Chem.* **2017,** *1*, 113−115.

5. Sicilia, V.; Forniés, J.; Casas, J. M.; Martín, A.; López, J. A.; Larraz, C.; Borja, P.; Ovejero, C.; Tordera, D.; Bolink, H. J., Highly Luminescent Half-Lantern Cyclometalated Platinum(II) Complex: Synthesis, Structure, Luminescence Studies, and Reactivity. *Inorg. Chem.* **2012,** *51*, 3427-3435.

6. Sicilia, V.; Borja, P.; Casas, J. M.; Fuertes, S.; Martín, A., Selective Synthesis of new half-Lantern Benzoquinolate Platinum Complexes. DFT and Photophysical Studies on the Platinum (II,II) Derivative. *J. Organomet. Chem.* **2013,** *731*, 10-17.

7. Forniés, J.; Sicilia, V.; Borja, P.; Casas, J. M.; Díez, A.; Lalinde, E.; Larraz, C.; Martín, A.; Moreno, M. T., Luminescent benzoquinolate-Isocyanide platinum(II) Complexes: Effect of Pt···Pt and π-π Interactions on their Photophysical properties. *Chem. Asian J.* **2012,** *7*, 2813-2823.

8. Doerrer, L. H., Steric and Electronic Effects in Metallophilic Double Salts. *Dalton Trans.* **2010,** *39*, 3543-3553.
